# Artificial intelligence-based characterization of multi-organ ultrasound congestion across the heart failure Spectrum

**DOI:** 10.1093/ehjimp/qyag036

**Published:** 2026-03-04

**Authors:** Lavinia Del Punta, Giacomo Aru, Alina Sirbu, Nicolò De Biase, Stefano Taddei, Giuseppe Prencipe, Stefano Masi, Nicola Riccardo Pugliese

**Affiliations:** Department of Clinical and Experimental Medicine, University of Pisa, Via Roma 67, Pisa 56124, Italy; Computer Science Department, University of Pisa, Largo Bruno Pontecorvo 3, Pisa, Italy; Department of Computer Science and Engineering, University of Bologna, Via Mura Anteo Zamboni 7, Bologna 40126, Italy; Department of Clinical and Experimental Medicine, University of Pisa, Via Roma 67, Pisa 56124, Italy; Department of Clinical and Experimental Medicine, University of Pisa, Via Roma 67, Pisa 56124, Italy; Department of Computer Science and Engineering, University of Bologna, Via Mura Anteo Zamboni 7, Bologna 40126, Italy; Department of Clinical and Experimental Medicine, University of Pisa, Via Roma 67, Pisa 56124, Italy; Department of Clinical and Experimental Medicine, University of Pisa, Via Roma 67, Pisa 56124, Italy

**Keywords:** ultrasound-defined congestion, multi-organ ultrasound, artificial intelligence, heart failure spectrum

## Abstract

**Aims:**

To investigate, using artificial intelligence (AI), the relationships between ultrasound (US)-defined systemic congestion and demographic, echocardiographic, and biohumoral parameters across the heart failure (HF) spectrum.

**Methods and results:**

A total of 1588 subjects (651 Stage A–B, 376 HF with reduced left ventricular ejection fraction [HFrEF, <50%], and 561 HF with preserved ejection fraction [HFpEF, ≥50%]) underwent comprehensive clinical evaluation, laboratory testing, echocardiography, and US assessment of congestion, including inferior vena cava (IVC), lung ultrasound (LUS), renal venous flow (RVF), portal venous flow (PVF), and hepatic venous flow (HVF). Assessment of IVC, LUS, and RVF was available in the entire cohort, whereas HVF and PVF were performed in 359 and 289 patients, respectively. Overall, 856 patients had no US signs of congestion, 458 had one US sign, and 274 had ≥2 US signs (multi-organ congestion). AI-based predictive models were developed for each site of congestion and for multi-organ congestion using a 3-item model (IVC, LUS, RVF). Congestion-related features clustered into four domains: medical history, biohumoral variables, left heart morphology and function, and right heart and pulmonary circulation. The 3-item model identified mitral annular systolic velocity, systolic and diastolic pulmonary artery pressure, triglycerides, left atrial volume index, diabetes, and treatment with furosemide or angiotensin-converting enzyme inhibitors/angiotensin II receptor blockers as key predictors of multi-organ congestion (area under the curve = 0.79).

**Conclusion:**

AI-assisted integration of multi-organ US characterizes congestion as a multidimensional phenotype beyond conventional clinical assessment and biomarkers across the HF spectrum.

## Graphical Abstract

**Graphical Abstract:**
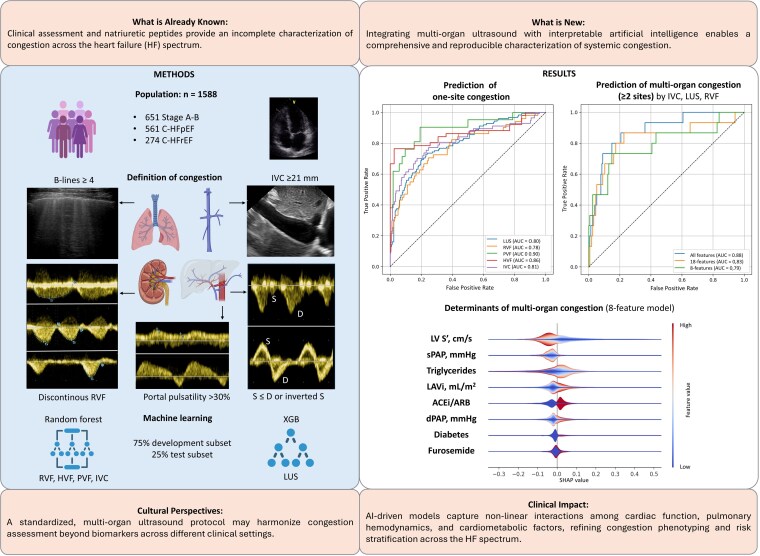
A model for each site of congestion (one-site models for IVC, LUS, RVF, HVF, PVF) and a 3-item model (IVC, LUS and RVF) were built integrating routine clinical, laboratory, and echocardiographic to predict one-site and multi-organ (≥2 US signs) congestion respectively. The minimal set of input features was selected for the 3-item model. Shapley additive explanations violin plot illustrates the direction and magnitude of each variable’s impact on the prediction of multisite congestion. ACEi/ARB: angiotensin-converting enzyme inhibitors/angiotensin II receptor blockers; AI: artificial intelligence; AUC = area under the receiver operating characteristic curve; dPAP = diastolic pulmonary artery pressure; HF = heart failure; HFpEF = heart failure with preserved ejection fraction; HFrEF = heart failure with reduced ejection fraction; HPV = hepatic venous flow; IVC = inferior vena cava; LAVi = left atrium volume index; LUS = lung ultrasound; LV S′ = left ventricle systolic mitral annulus tissue velocity; PVF = portal venous flow; RVF = renal venous flow; sPAP = systolic pulmonary artery pressure, US = ultrasound; XBG = extreme gradient boosting.

## Introduction

Congestion, defined as extracellular fluid accumulation, is a hallmark of heart failure (HF), driving symptoms, poor quality of life, and prognosis^1^. This ominous hemodynamic status results from salt and water retention, which leads to increased ventricular filling pressures, elevated pressures in the pulmonary and/or systemic venous compartments, and ultimately fluid leakage into the extravascular space. Despite its crucial role in defining HF diagnosis, progression, and outcomes, evaluating congestion remains challenging.^2,3^ Clinical assessment often fails to detect early stages of hemodynamic deterioration, and natriuretic peptides have several limitations, as they are influenced by various confounders (e.g. age, body mass index, renal function, atrial fibrillation). Ultrasound (US) offers a non-invasive, objective, and real-time evaluation of fluid overload.^4^ This approach is expanding in the setting of acute HF, as it can detect subtle hemodynamic changes and guide diuretic or fluid management. In particular, the presence of US congestion in ≥2 different sites is associated with poor outcomes and shows greater diagnostic accuracy than clinical examination and natriuretic peptide levels.^5,6^ Such an approach has also shown promising results in improving the diagnostic and prognostic stratification of patients at risk or with an established diagnosis of HF.^7–9^ Artificial intelligence (AI) has already been proposed to identify phenotypes of congestion in acute HF,^10^ as it can analyze and integrate vast amounts of data and account for intricate multidimensional interactions. The present paper aims to develop AI-based models to gain more insight into the relationships between US multi-organ congestion and clinical, biohumoral, and echocardiographic variables routinely obtained during a standard evaluation in outpatients with cardiovascular risk factors or an established diagnosis of HF. Combining AI with a rigorous, reproducible, and objective assessment of congestion could expand knowledge of fluid overload and help physicians focus on specific conditions linked to multi-organ congestion, tailoring the diagnostic approach, follow-up, and therapy.

## Methods

### Study population

Between January 2017 and July 2025, we prospectively enrolled consecutive patients referred for dyspnea at the University Hospital of Pisa (Italy, *n* = 1780). According to the American College of Cardiology/American Heart Association (ACC/AHA) HF staging system, patients were classified as Stage A (asymptomatic subjects with cardiovascular risk factors), Stage B (structural heart disease without signs or symptoms of HF), or Stage C (clinically overt HF). All Stage C patients had a prior diagnosis of HF or were newly diagnosed with HF by an independent physician blinded to the study protocol. A new diagnosis of HF required at least two typical HF signs or symptoms, including third heart sound, pulmonary rales, jugular venous distension, hepatomegaly, peripheral edema, or lung congestion on lung US or chest X-ray. HF with reduced LV ejection fraction (HFrEF) was defined by a LVEF <50%, while HF with preserved ejection fraction (HFpEF) required a LVEF ≥50%, N-terminal pro-B-type natriuretic peptide (NT-proBNP) > age-adjusted rule-in thresholds (≥125 pg/mL, ≥250 pg/mL, and ≥500 pg/mL for <50 years, 50–74 years, and ≥75 years, respectively),^11^ and the additional presence of relevant structural heart disease or diastolic dysfunction.^12^ Symptoms and quality of life were evaluated using the New York Heart Association (NYHA) class and the Kansas City Cardiomyopathy Questionnaire (KCCQ). All patients were hemodynamically stable at recruitment and had no regular workout routine. Patients with moderate or greater left-sided valve disease were excluded (*n* = 192).

The study complied with the requirements of the Declaration of Helsinki. The local ethics committee approved the protocol, and written informed consent was obtained from all patients. All authors had full access to the data, took responsibility for its integrity, contributed to the manuscript, and agreed to this report as written.

### Study protocol

The study protocol is part of a standardized workup in a dedicated dyspnea clinic.^13^ All measurements were performed in a quiet room with a stable room temperature. No meals, caffeine, or smoking were permitted for 3 h before the evaluation. Therapy remained unchanged during the whole protocol. The evaluation included a comprehensive clinical assessment, blood tests, a 12-lead ECG, and an US examination. All patients underwent spirometry; chronic obstructive pulmonary disease was defined as forced expiratory volume in 1 s [FEV1]/forced vital capacity [FVC] < 0.70, and more than moderate airflow obstruction as FEV1 < 50% of predicted FEV1.

### Laboratory evaluation

Patients were instructed to fast overnight and refrain from taking any medications before blood and urine sampling on the morning of the tests. A more detailed description of the laboratory protocol is provided in the Supplementary data online, Appendix.

### Baseline echocardiography

All patients underwent a standard transthoracic echocardiographic examination using the LISENDO 880 (Hitachi Medical Systems, Tokyo, Japan), in accordance with international recommendations.^14^ The echocardiographic protocol has been previously described^15^ and is provided in the Supplementary data online, Appendix.

### Multi-organ congestion

Multi-organ congestion was assessed using an integrated US approach. A linear transducer was used for B-lines evaluation, while a phased-array transducer was used for all US signs of venous congestion. Color Doppler imaging was set to low-flow velocities (<20 cm/s) for venous flow assessment, and measurements were performed during a breath-hold to mitigate distortions. The ECG signal synchronized venous flow signals with the cardiac cycle.

#### Inferior vena cava (IVC)

With the patient in the supine position, the maximum IVC diameter during the respiratory cycle was measured in the subcostal long-axis view, located between 1 and 3 cm before the IVC merged with the right atrium. The IVC collapse was visually estimated as 50 or <50% following deep inspiration (a brief sniff).

#### Renal venous flow (RVF)

The patient was in the left lateral decubitus position for interlobar RVF analysis, and the right kidney was scanned longitudinally. We used a semi-quantitative assessment, distinguishing continuous (normal conditions), discontinuous pulsatile or biphasic (worsening congestion), and monophasic (most severe cases) RVF.

#### Liver ultrasound

HVF and PVF were evaluated with the patient supine in the mid-to-posterior axillary line with the probe marker directed toward the patient’s head. We used a quantitative assessment of HVF, evaluating the systolic (S) and diastolic (D) wave amplitude to distinguish S > D (normal conditions) vs. S ≤ D or reversed S as signs of worsening congestion. The severity of alterations in flow dynamics in the portal vein was assessed using the pulsatility fraction, calculated as the difference between the maximal and minimal PVF velocities, divided by the maximal PVF velocity, and expressed as a percentage.

#### Lung ultrasound (LUS)

B-lines were measured in parallel orientation (transverse) to the ribs at an imaging depth of ∼15–18 cm using an eight-region scan. In each region, B-lines were counted individually if distinguishable; if confluent, we estimated their number by the percentage of screen space occupied, divided by 10 (up to a max of 10 B-lines/region). The sum of B-lines across the eight scanning regions yielded a score indicating the extent of extravascular lung water.

We defined congestion by US as a discontinuous RVF, PVF pulsatility ≥30%, HVF S ≤ D or reversed S, IVC ≥21 mm, and B-lines above or equal to the lower boundary of the highest tertile (≥4). We further classified the population according to the number of US signs of congestion, distinguishing between no signs, 1 sign, or ≥2 signs (i.e. multi-organ congestion), as previously described^7^ (*Graphical Abstract*).

### Conventional statistical analysis

Categorical variables are presented as numbers and percentages and compared using Pearson’s Chi-square test or Fisher’s exact test, as appropriate. Continuous variables are expressed as median and interquartile range and compared using the Student’s *t*-test or the Mann–Whitney *U* test. Group differences were assessed using one-way analysis of variance or the Kruskal–Wallis test, with a Bonferroni correction applied for multiple comparisons (significance threshold: *P* < 0.01). All tests were two-sided, and statistical significance was defined as *P* < 0.01.

### AI-driven model development

We developed a custom machine learning pipeline to predict US-defined congestion at individual sites (one-site models for IVC, LUS, RVF, HVF, and PVF) and to identify multi-organ congestion using a 3-item model including IVC, LUS, and RVF, as these measures were available for all patients. The dataset was randomly split into a development set (75%) and an independent test set (25%). Model selection and optimization were conducted exclusively within the development set using a 6-fold cross-validation framework. Variables with excessive missingness (>20%) and those with high collinearity (pairwise correlation coefficient >0.8) were excluded. Missing values were imputed using a K-nearest neighbors algorithm, and class imbalance was addressed using Synthetic Minority Over-sampling. Both procedures were applied only within training folds to prevent data leakage. After preliminary analyses, Logistic Regression, Random Forest, and Extreme Gradient Boosting were retained, while Support Vector Machines and Multilayer Perceptrons were excluded due to suboptimal performance. Hyperparameter tuning was performed using a 500-iteration random search. Model performance was evaluated using the area under the receiver operating characteristic curve (AUC) and the F1 score, which reflects the harmonic mean of precision and recall and accounts for class imbalance. As conventional feature selection methods proved unstable, we implemented a SHapley Additive exPlanations (SHAP)-based reduction strategy. Variables were ranked by SHAP values derived from training data, and models were iteratively retrained on feature subsets of increasing size. Model selection prioritized the best average cross-validation performance combined with minimal variability in AUC and F1 score. To enhance clinical applicability, we retained the best-performing configuration and, in addition, a restricted model with a maximum of 10 variables, and compared it with both the complete and optimal unrestricted models. Calibration of the final restricted 3-item model was assessed using calibration intercept, slope, and Brier score, and the distribution of predicted probabilities was examined across the 0–1 range.

## Results

### Study population characteristics

The population cohort consisted of 1588 subjects (development *n* = 1191; test *n* = 397). Demographic, clinical, and laboratory characteristics according to the number of sites of US congestion are reported in *Table 1*, and by ACC/AHA HF stages in Supplementary data online, *Table S1*. Patients with increasing congestion were older, had lower body mass index, and more often displayed atrial fibrillation and previous stroke. NYHA class increased with worsening congestion, while KCCQ reduced progressively. Multi-organ congestion was more frequent in patients with an established diagnosis of HF, despite higher use of loop diuretics, mineralocorticoid receptor antagonist, and sodium glucose cotransporter 2 inhibitors.

**Table 1:** Population characteristics

Variable	No US signs of congestion (*n* = 856)	1 US sign of congestion (*n* = 458)	≥2 US signs of congestion (*n* = 274)
**Demographics**			
Age, years	70 (58–78)	75 (63–81)^a^	79 (74–84)^a,b^
Males	534 (62)	296 (65)	170 (62)^c^
BMI, kg/m^2^	26 (23–29)	26 (24–29)	24 (22–28)^a,b^
Arterial hypertension	561 (67)	322 (70)	186 (68)
Diabetes mellitus	296 (23)	122 (27)	74 (27)
Dyslipidemia	467 (55)	291 (65)	159 (58)^d^
Smoke	202 (24)	127 (28)	80 (29)^c^
AHA/ACC stages			
A-B	453 (53)	165 (36)	33 (11)^c^
C-HFpEF	220 (40)	180 (32)	161 (28)
C-HFrEF	183 (48)	113 (30)	80 (22)
History of atrial fibrillation	154 (18)	139 (30)	172 (63)^c^
Atrial fibrillation/flutter	66 (8)	75 (17)	132 (51)^c^
Stroke/TIA	53 (6)	31 (7)	30 (11)^d^
CAD	169 (20)	102 (23)	60 (22)
Coronary revascularization	150 (18)	92 (23)	54 (20)
Previous MI	108 (13)	70 (15)	44 (16)
COPD	26 (3)	13 (2)	54 (2)
FEV1 < 50%	3 (0)	4 (2)	4 (1)
**Clinical evaluation**			
Heart rate, beats/min	75 (65–85)	75 (65–85)	75 (65–85)
Systolic blood pressure, mmHg	130 (120–145)	130 (140)	128 (113–140)^a^
Diastolic blood pressure, mmHg	80 (70–85)	80 (70–80)	75 (7–85)
Oxygen saturation, %	98 (97–99)	98 (97–99)	98 (97–99)
NYHA classification			
Class I	463 (54)	185 (41)	75 (27)^c^
Class II	310 (36)	203 (44)	136 (50)
Class III	82 (10)	68 (15)	63 (23)
KCCQ score, %	74 (59–87)	74 (59–86)	67 (50–80)^a,b^
**Therapy**			
Beta-blockers	488 (52)	295 (64)	208 (76)^c^
DHP CCB	171 (20)	107 (23)	36 (13)^d^
Non-DHP CCB	13 (2)	9 (2)	4 (2)
Amiodarone	28 (3)	17 (6)	17 (6)
Digoxin	34 (4)	21 (5)	20 (7)
ACEi or ARB	457 (53)	271 (59)	143 (52)
MRA	199 (23)	131 (29)	130 (48)^c^
ARNI	890 (9)	53 (12)	23 (8)
Loop diuretics	291 (34)	215 (47)	217 (79)^c^
Loop diuretics daily dose	0 (0–25)	0 (0–25)^a^	25 (12.5–50)^a,b^
Thiazides/thiazide-like diuretics	110 (13)	56 (12)	26 (10)
SGLT2i	57 (7)	47 (10)	80 (29)^c^
ICD	62 (7)	41 (9)	28 (10)
CRT	31 (4)	27 (6)	22 (8)
LAMA	31 (4)	28 (6)	16 (6)
LABA	43 (5)	32 (7)	21 (8)
Inhaled corticosteroids	42 (5)	22 (5)	21 (8)
**Blood tests**			
WBC, cells/µL	6.33 (5.30–7.63)	6.05 (5.10–7.27)	6.29 (5.11–7.45)
NLR	2.18 (1.71–2.96)	2.39 (1.75–3.47)	2.85 (2.00–4.04)^a,b^
Hemoglobin, g/dL	13.5 (12.5–14.4)	13.3 (12.2–14.5)	12.7 (11.5–13.9)^a,b^
RDW, %	13.5 (14.4–13.5)	13.3 (12.2–14.5)	12.7 (11.5–13.9)^a,b^
TSAT, %	23 (18–29)	23 (17–29)	18 (13–24)^a,b^
Iron, μmol/L	78 (61–95)	75 (58–95)	65 (50–81)^a,b^
Ferritin, ng/mL	116 (57–202)	100 (57–202)	90 (49–196)
Creatinine, mg/dL	0.96 (0.81–1.15)	0.99 (0.81–1.21)	1.13 (0.91–1.43)^a,b^
eGFR, mL/min/1.73 m^2^	72 (56–85)	68 (54–83)	58 (43–74)^a,b^
ACR, mg/dL	9 (5–27)	10 (4–31)	16 (5–51)
Na^+^, mEq/L	141 (139–142)	140 (139–142)	141 (139–142)
K^+^, mEq/L	4.3 (3.9–4.6)	4.3 (4.0–46)	4.3 (4.0–4.6)
Albumin, g/dL	4.2 (4.0–6.4)	4.3 (4.0–4.5)	4.2 (4.0–4.5)
HbA1c, mmol/mol	40 (37–45)	40 (37–45)	42 (38–47)^a^
Total cholesterol, mg/dL	166 (138–196)	158 (131–187)	149 (124–176)^a^
LDL, mg/dL	95 (72–119)	87 (66–113)	84 (60–105)^a^
HDL, mg/dL	50 (42–71)	51 (41–61)	50 (42–61)
Triglycerides, mg/dL	96 (74–133)	93 (72–125)	82 (67–107)^a^
Uric acid, mg/dL	5.4 (4.4–6.4)	5.6 (4.6–6.70)	5.8 (4.7–7.4)^a^
hs-CRP, mg/L	0.14 (0.01–0.32)	0.14 (0.02–0.32)	0.18 (0.10–0.50)^a^
NT-proBNP, pg/mL	288 (109–781)	462 (190–1233)^a^	1521 (625–2739)^a,b^
hs-Troponin T, pg/mL	16 (10–27)	16 (10–26)	17 (10–29)

Values are *n* (%) or median (25th quartile, 75th quartile).

^a^ *P* < 0.001 vs. no US signs

^b^p < 0.001 vs. 1 US sign.

^c^ *P* < 0.001 for *χ*^2^ tests;

^d^ *P* 0.005

ACEi: angiotensin-converting enzyme inhibitor; ACR: albumin-to-creatinine ratio; AHA/ACC: American College of Cardiology/American Heart Association; ARB: angiotensin receptor blocker; ARNI: angiotensin receptor neprilysin inhibitor; BMI: body mass index; CAD: coronary artery disease; CRT: cardiac resynchronization therapy; DHP CCB: dihydropyridine calcium channel blocker; eGFR: estimated glomerular filtration rate; HbA1c: glycated hemoglobin; HFpEF: heart failure with preserved ejection fraction; HFrEF: heart failure with reduced ejection fraction; hs-CRP: high sensitivity C-reactive protein; hs-Troponin T: high sensitivity Troponin T; ICD: implantable cardioverter defibrillator; MI: myocardial infarction; MRA: mineralocorticoid receptor antagonist; NYHA: New York Heart Association; NLR: neutrophil-to-lymphocyte ratio NT-proBNP: N-terminal prohormone of brain natriuretic peptide; RDW: red cell distribution width; SGLT2i: sodium glucose co-transporter 2 inhibitors; TIA: transient ischemic attack, TSAT: transferrin saturation; WBC: white blood cell.

### Laboratory evaluation

NT-proBNP levels increased progressively with the number of US signs of congestion. Those with multi-organ congestion showed higher inflammation markers [neutrophil-to-lymphocyte ratio, high-sensitivity C-reactive protein, uric acid, and red cell distribution width (RDW)] and serum creatinine, with lower hemoglobin, iron, and transferrin saturation (TSAT) levels (all *P* < 0.01), compared with patients with no or 1 US sign of congestion (***Table 1***).

### Echocardiography

Patients with ≥2 US signs of congestion showed more pronounced LV impairment: lower LVEF, reduced tissue Doppler mitral annulus systolic velocity (LV S′), and greater global longitudinal strain impairment, compared with those without congestion (***Table 2***). Markers of diastolic dysfunction—including higher mitral E velocity, increased E/A ratio, and higher E/e′—worsened in parallel with congestion severity. Left atrial remodeling was evident, with significantly larger left atrial volume index (LAVi) and markedly reduced reservoir and booster strain in the group with ≥2 US signs. Right-sided chambers and pulmonary pressures were also affected: individuals with more advanced congestion demonstrated larger right atrial area, higher systolic (sPAP) and diastolic pulmonary arterial pressure (dPAP), and a substantial reduction in right ventricle (RV) systolic performance [i.e. lower tricuspid annular plane systolic excursion (TAPSE)] and RV-pulmonary coupling (i.e. TAPSE/sPAP). Consistently, tricuspid regurgitation severity increased stepwise, with severe TR present in more than one-third of patients with ≥2 US signs of congestion.

**Table 2:** Baseline echocardiography

Variable	No US signs of congestion (*n* = 856)	1 US sign of congestion (*n* = 458)	≥2 US signs of congestion (*n* = 274)
**Left ventricle**			
WMSI	1.0 (1.0–1.0)	1.0 (1.0–1.4)	1.0 (1.0–1.5)
LVMi, g/m^2.7^	111 (93–133)	119 (100–140)^a^	112 (91–142)
RWT	0.37 (0.32–0.42)	0.36 (0.36.0.42)	0.38 (0.32–0.44)
LVEDVi, mL/m^2^	69 (56–87)	75 (60–95)^a^	71 (56–94)
LV ejection fraction, %	62 (51–67)	60 (48–64)	58 (45–65)^a^
Average S′, cm/s	8.0 (6.4–9.5)	6.0 (5.3–8.0)^a^	6.0 (5.5–7.5)^a^
LV GLS, %	16 (12–19)	15 (11–18)	14 (10–17)^a^
SV, mL/beat	61 (50–76)	57 (48–72)	52 (41–64)^a,b^
CO, L/min	4.7 (3.9–5.9)	4.8 (3.8–5.6)	4.3 (3.0–5.6)
Mitral E, cm/s	80 (63–100)	90 (70–120)^a^	120 (85–170)^a,b^
Mitral A, cm/s	90 (70–110)	85 (65–110)	85 (60–115)
Mitral E/A	0.86 (0.67–1.17)	1 (0.72–1.37)^a^	1.3 (0.8–2.3)^a,b^
Average *E*/*e*′ LA reservoir strain/*E*/*e*′	10 (7–13) 2.60 (1.51–4.49)	11 (9–16)^a^ 1.79 (0.95–3.10)^a,b^	13 (9–20)^a,b^ 0.97 (0.60–1.80)^a,b^
**Left atrium**			
LAVi, mL/m^2^	34 (27–43)	40 (32–51)^a^	51 (39–66)^a,b^
LA reservoir strain, %	26 (19–36)	22 (15–30)^a^	14 (9–20)^a,b^
LA booster strain, % (SR only)	14 (9–18)	12 (8–16)^a^	9 (4–13)^a,b^
LA reservoirs strain/*E*/*e*′	2.60 (1.51–4.49)	1.79 (0.95–3.10)^a^	0.97 (0.59–1.78)^a,b^
**Right ventricle and pulmonary circulation**			
RA area, cm^2^	19 (15–30)	19 (16–25)	26 (21–34)^a,b^
TAPSE, mm	21 (18–23)	20 (18–23)	18 (17–21)^a,b^
sPAP, mmHg	29 (23–37)	34 (26–45)^a^	49 (37–60)^a,b^
dPAP, mmHg	9 (7–11)	10 (8–13)^a^	15 (10–20)^a,b^
TAPSE/sPAP, mm/mmHg	0.72 (0.55–0.90)	0.60 (0.43–0.80)^a^	0.38 (0.29–0.54)^a,b^
TR severity			
Mild	723 (84)	320 (70)	89 (32)^c^
Moderate	51 (6)	61 (13)	55 (20)
Severe	6 (1)	17 (4)	103 (38)
EROA-TR^d^	0.27 (0.22–0.37)	0.22 (0.21–0.22)	0.30 (0.29–0.31)
**Congestion assessment**			
IVC, mm	15 (15–18)	15 (15–19)^a^	22 (18–25)^a,b^
IVC ≥21 mm	0 (0)	78 (17)	162 (59)^c^
IVC collapse <50%	17 (2)	27 (6)	108 (39)^c^
B-lines	0 (0–2)	6 (4–11)^a^	9 (5–18)^a,b^
B-lines ≥ 4	0 (0)	307 (67)	197 (72)^c^
B-lines cardiogenic	157 (18)	97 (21)	105 (39)^c^
RVF pattern			
Continuous	402 (100)	403 (88)	77 (28)^c^
Discontinuous: pulsatile	0 (0)	41 (9)	96 (35)
Discontinuous: biphasic	0 (0)	9 (2)	41 (15)
Discontinuous: monophasic	(0)	5 (1)	60 (22)
Hepatic vein			
S > D	78 (100)	52 (56)	25 (13)^c^
S ≤ D	0 (0)	33 (36)	57 (30)
Systolic flow reversal	0 (0)	7 (8)	107 (57)
Portal vein pulsatility index	17 (11–22)	19 (13–25)	36 (21–55)^a,b^
Pulsatility index >30	0 (0)	14 (15)	70 (59)^c^

Values are *n* (%) or median (25th quartile, 75th quartile).

^a^ *P* < 0.001 vs. no signs of congestion.

^b^ *P* < 0.001 vs. 1 sign of congestion.

^c^ *P* < 0.001 for *χ*^2^ tests.

^d^Only in at least moderate tricuspid regurgitation.

HVF was performed in *n* = 359 and PVF in *n* = 289.

CO: cardiac output; EDVi: end-diastolic volume index; EROA: effective regurgitant orifice area; IVC: inferior vena cava; LA: left atrium: LAVi: left atrial volume index; LV: left ventricle; LVEDVi: left ventricle end-diastolic volume index; LV GLS: left ventricle global longitudinal strain; LVMi: left ventricular mass index; RVF: renal venous flow; RWT: relative wall thickness; sPAP: systolic pulmonary artery pressure; SR, sinus rhythm; SV: stroke volume; TAPSE: tricuspid annular plane systolic excursion; TR: tricuspid regurgitation; WMSI: wall motion score index.

### Evaluation of congestion

Assessment of IVC, LUS, and RVF was available for the whole population, while HVF was performed in *n* = 359 and PVF in *n* = 289. The presence of at least 1 US sign of congestion was more common in patients with HF (HFpEF *n* = 341, 60%; HFrEF *n* = 193, 52%), but US assessment revealed at least 1 sign of congestion in 47% (*n* = 198) of patients in Stages A–B (*n* = 198, 47%; *P* < 0.001; Supplementary data online, *Table S2*). In contrast, differences in the number of congestive sites were not significant between HFrEF and HFpEF (*P* = 0.07). Of note, the percentage of B-lines with a likely non-cardiogenic origin was higher in patients with Stages A–B than in those with established HF.

### AI-driven prediction of one-site congestion

**Figure 1:**
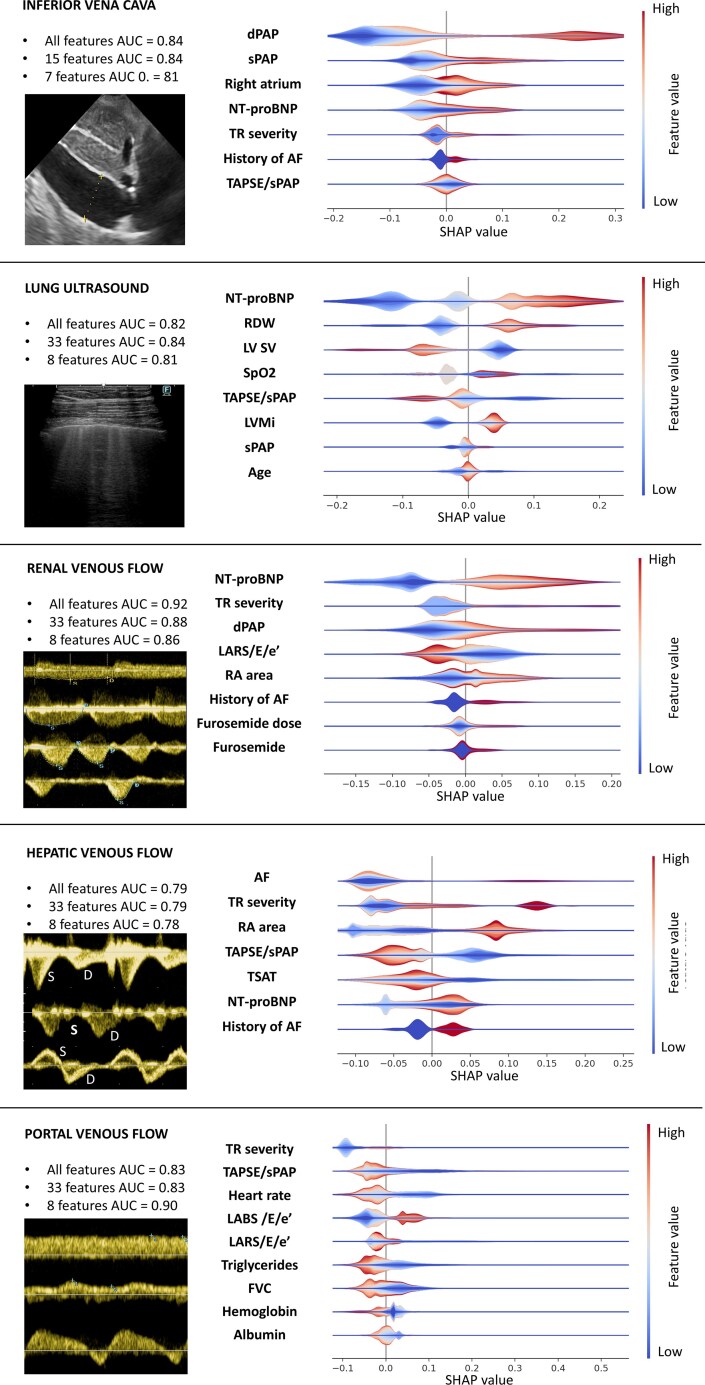
Performance and interpretability of one-site congestion models. Shapley additive explanations violin plot illustrating the direction and magnitude of each variable’s impact on the prediction of one-site congestion. AF: atrial fibrillation; dPAP: diastolic pulmonary artery pressure; LARS: left atrium reservoir strain; LABS: left atrium booster strain; LAVi: left atrial volume index; LV SV: left ventricle stroke volume; LVMi: left ventricular mass index; NT-proBNP: N-terminal prohormone of brain natriuretic peptide; RA: right atrium; RDW: red cell distribution width; sPAP: systolic pulmonary artery pressure; SpO2: oxygen saturation; TAPSE: tricuspid annular plane systolic excursion; TSAT: transferrin saturation; TR: tricuspid regurgitation.

The list of available variables is shown in Supplementary data online, *Table S3*. The one-site models achieved good performance on test data, as summarized in ***Figure 1***. The results of the iterative training for the one-site models are shown in the Supplementary data online, *Figures S1*–S*5*. The restricted model (i.e. <10 variables) performed slightly worse than the best model at each US congestion site, except PVF. Nevertheless, AUCs were satisfactory across all models, suggesting a good compromise between performance and the number of variables.

The parameters associated with congestion varied across the models, but they can be summarized into four domains: medical history, biohumoral variables, left heart morphology and function, and right heart and pulmonary circulation. The distributions of those parameters across models are shown in ***Figure 1*** and *V*. The LUS model was also tested separately in patients having cardiogenic and non-cardiogenic B-lines, with AUCs of 0.81 and 0.72, respectively.

### AI-driven prediction of multi-organ congestion

**Figure 2:**
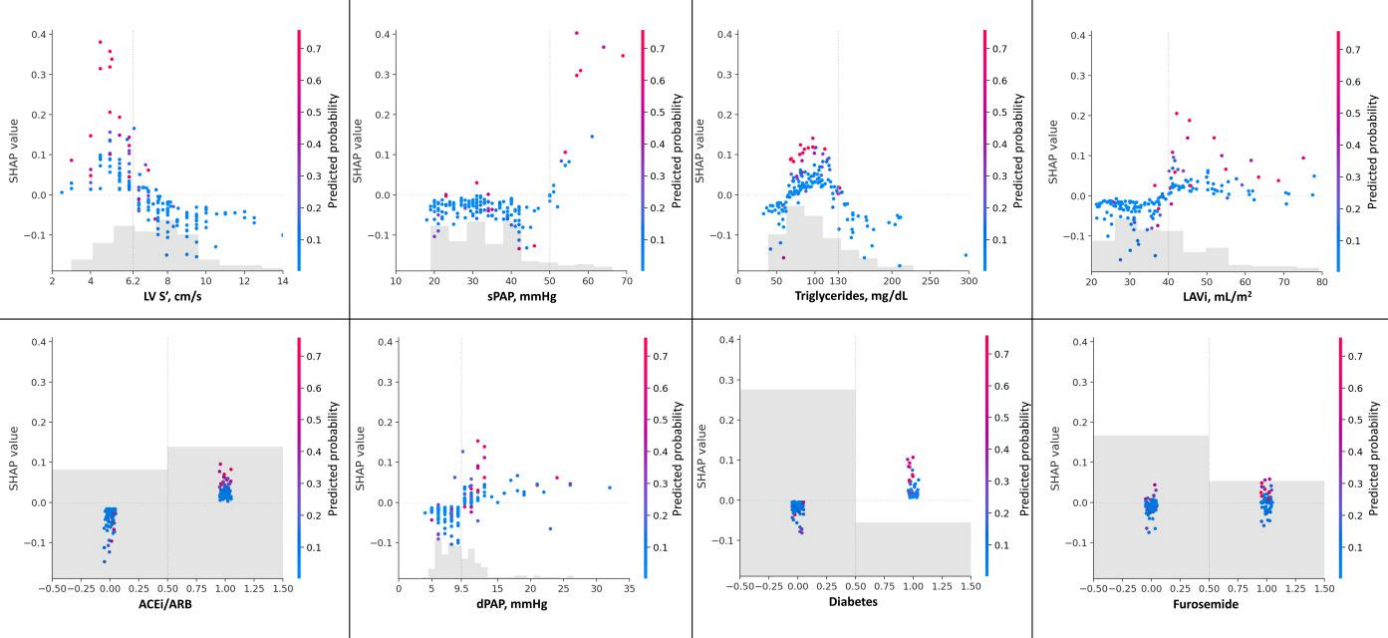
Performance and interpretability of the 3-item model (IVC, LUS, RVF) for predicting multi-organ congestion. Each panel shows the relationship between the feature value (*x*-axis) and its SHAP value (*y*-axis), indicating the feature’s contribution to the model output. Points are colored by the predicted probability of the positive class. ACEi: angiotensin-converting enzyme inhibitor; ARB: angiotensin receptor blocker; dPAP: diastolic pulmonary artery pressure; IVC: inferior vena cava; LAVi: left atrial volume index; LUS: lung ultrasound; LV: left ventricle; RVF: renal venous flow; sPAP: systolic pulmonary artery pressure.

The 3-item (IVC, LUS, RVF) model predicted congestion in ≥2 sites, with AUC 0.88 including all variables, 0.83 with 18 variables, and 0.79 in the restricted model with eight variables (*Graphical Abstract*). LV S’ was the most influential feature, followed by sPAP, plasmatic levels of triglycerides, LAVi, therapy with angiotensin-converting enzyme inhibitors (ACEi) or angiotensin II receptor blockers (ARBs), dPAP, diabetes, and the use of furosemide (***Figure 2***). Importantly, SHAP analysis identifies thresholds at which risk factors become detrimental, i.e. where they start to increase the model’s congestion probability.

The model showed moderate calibration performance. The calibration curve deviated from the ideal line across several bins, with a Brier score of 0.072 (see Supplementary data online, *Figure S6A*), and the predicted probabilities were concentrated toward lower values, consistent with class imbalance between the groups with and without multi-organ congestion (see Supplementary data online, *Figure S6B***)**. The 3-item model demonstrated consistent robustness in the C-HFrEF (AUC 0.90) and C-HFpEF (AUC 0.98) subgroups.

## Discussion

This study explored the association between US multi-organ congestion (evaluated by IVC, LUS, RVF, HVF, PVF) and clinical, biohumoral, and echocardiographic characteristics in patients at risk of or with established HF, applying AI to detect complex, non-linear relations between variables. A 3-item model based on IVC, LUS, and RVF identified LV S’, sPAP, triglycerides, LAVi, ACEi/ARB, dPAP, diabetes, and therapy with furosemide as predictors of multi-organ congestion (i.e. ≥2 sites).

### Multi-organ congestion

Increasing evidence suggests that US-based congestion provides a more accurate characterization of patients across the HF spectrum. Pellicori et al. found that more than 40% of clinically euvolemic outpatients with HF have at least one sign of US-detected congestion, and more than 10% have two.^8^ Cuthbert et al. further highlighted the role of US in redefining fluid overload, showing that subclinical US-congestion was present in 30% of patients with cardiovascular risk factors, but without a diagnosis of HF.^4^ In the present study, the multisite US approach confirmed subtle fluid overload in patients who did not meet HF guideline criteria. These findings suggest that implementing routine evaluation with multi-organ US-based congestion across the entire HF spectrum may refine risk stratification and hemodynamic management in patients at risk of, or with established HF. The multisite protocol proved feasible and time-efficient, and it could be easily integrated into routine evaluations in HF clinics, as it requires minimal training and demonstrates high reproducibility.^7^

With an ageing population and advances in medical care, clinicians are increasingly confronted with complex patients and multiple comorbidities. A multi-organ US-based assessment can help differentiate cardiogenic congestion from other conditions that produce isolated abnormalities, such as reduced venous compliance or venous dilation secondary to hydronephrosis, hepatic steatosis, or cirrhosis. Likewise, it enhances LUS interpretation by distinguishing B-lines arising from primary pulmonary pathology and identifying cardiogenic congestion in syndromes with clinical features overlapping HF.^16^

### AI-driven interpretation of multi-organ congestion

AI has been proposed to define congestion phenotypes in acute HF, relying on clinical signs of congestion, biohumoral variables, and echocardiography.^10^ The present study offers a more rigorous US-based definition of congestion, applied to a cohort of clinically stable patients at risk for or with established HF. The predictive models provide a coherent, physiologically grounded, and clinically interpretable representation of multi-organ congestion in HF. The many parameters derived across models underscore the multifaceted nature of congestion, a condition that cannot be fully captured by left ventricular function alone or NT-proBNP levels. Indeed, congestion reflects complex hemodynamic interactions involving the lungs and RV, as well as metabolic comorbidities and biohumoral markers of inflammation and frailty. Pulmonary hypertension and RV dysfunction are increasingly recognized as key determinants of symptom burden and adverse outcomes in HF.^17,18^ Their contribution in the models reflects a broader pathophysiological interplay in which elevated left-sided filling pressures propagate backward into the pulmonary circulation, ultimately exacerbating both pulmonary and systemic congestion.^19^ The presence of diabetes and triglycerides as congestion-related predictors aligns with growing evidence that cardiometabolic comorbidities are not merely concurrent conditions but are mechanistically intertwined with HF progression.^20^ Metabolic dysregulation contributes to endothelial dysfunction, microvascular inflammation, and impaired myocardial energetics, all of which can intensify systemic congestion^21–23^ Similarly, alterations in RDW and TSAT have been associated with multiple markers of cardiac dysfunction and HF severity, including natriuretic peptides, left ventricular end-diastolic pressure, and peak oxygen consumption.^15,24^ RDW reflects systemic inflammatory stress, impaired erythropoiesis, and neurohormonal activation, whereas reduced TSAT signals iron deficiency—an established driver of reduced exercise capacity and poor prognosis in HF.^15,24^

The 3-item model confirmed the findings from one-site models. SHAP analysis revealed LV S’ as the most influential predictor, with lower values strongly increasing the multi-organ congestion risk. Fluid overload occurs irrespective of an impaired LVEF,^25,26^ but the prominence of LV S’ in predicting congestion is consistent with its high sensitivity in describing longitudinal systolic function.^27,28^ LV S’ can be impaired in early stages of cardiac disease—even when conventional indices of systolic performance, including LVEF, remain within normal limits—and it is strongly associated with prognosis in HF.^28^ Similarly, higher sPAP, dPAP, and larger LAVi, all markers of elevated LV filling pressure,^29,30^ showed positive SHAP values, indicating a high likelihood of congestion. The inclusion of dPAP alongside parameters commonly measured to assess LV diastolic function, such as sPAP and LAVi, confirms the necessity for a more precise characterization of pulmonary hemodynamics, as suggested by the one-site models.^17^ The use of ACEi/ARBs or furosemide contributed slightly positively in the 3-item model, indicating a modest likelihood of congestion among patients on these medications, who are likely in a more advanced stage of disease. The diagnosis of diabetes was modestly associated with the increased probability of multi-organ congestion. Plasma triglyceride levels exhibited a heterogeneous, non-linear association with multi-organ congestion. Moderate-to-high triglyceride concentrations were associated with an increased likelihood of multi-organ congestion, whereas very high levels showed a neutral or even inverse association. This pattern implies that triglycerides influence the model less directly, functioning instead as a proxy for broader metabolic conditions rather than as an isolated determinant of congestion. Noteworthy, NT-proBNP was a predictor of congestion in the IVC, LUS, RVF, and HVF models, but its role was not confirmed in the 3-item model. Although NT-proBNP reflects systemic hemodynamic stress and neurohormonal activation related to congestion at individual sites, its incremental predictive value may be attenuated when a more comprehensive, multi-organ assessment of congestion is performed.

### Clinical perspective

**Figure 3:**
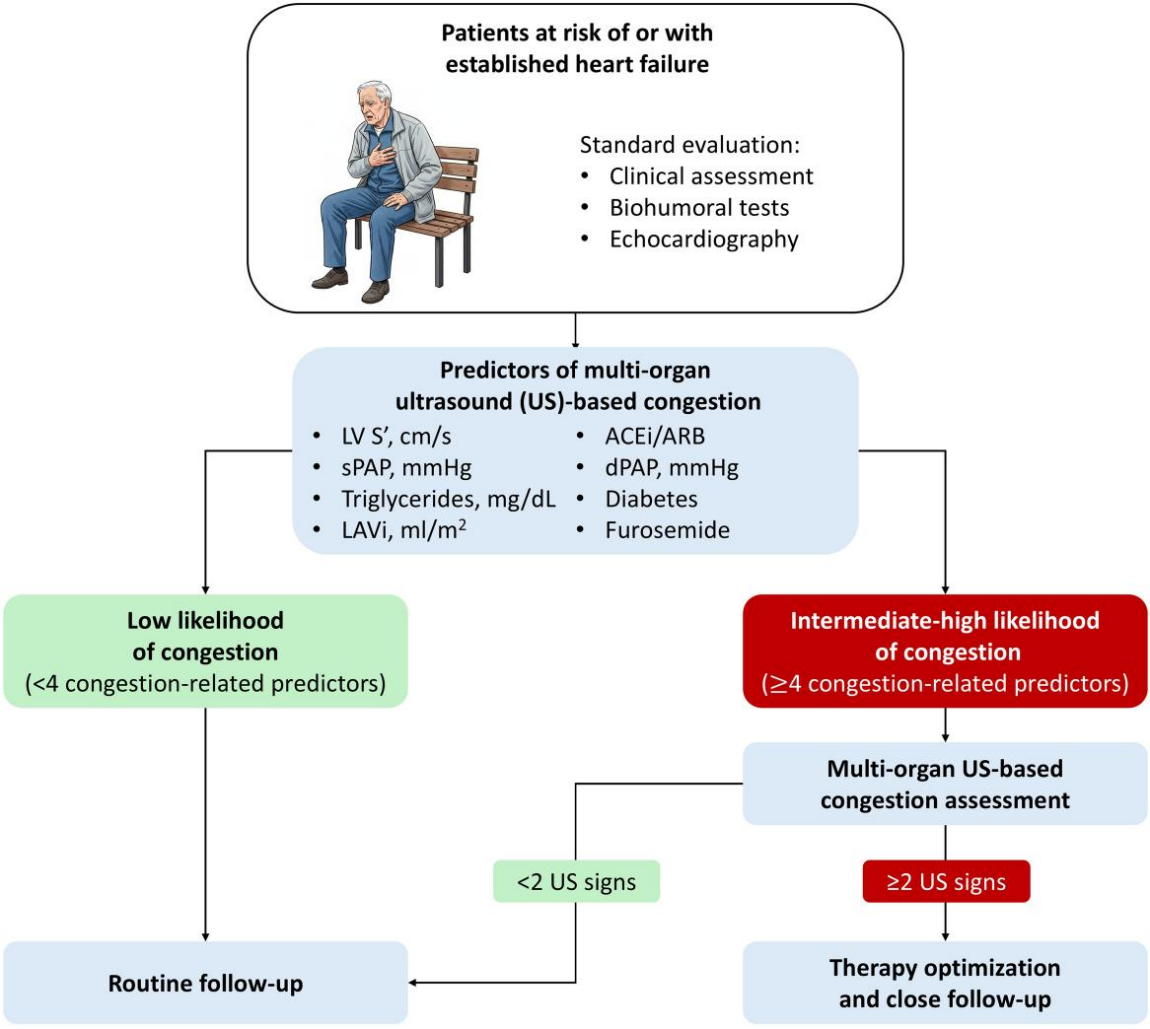
Hierarchical algorithm to integrate multi-organ ultrasound evaluation in clinical practice. ACEi: angiotensin-converting enzyme inhibitor; ARB: angiotensin receptor blocker; dPAP: diastolic pulmonary artery pressure; LAVi: left atrial volume index; LV: left ventricle; sPAP: systolic pulmonary artery pressure; US: ultrasound.

Adding IVC, LUS, and RVF evaluations to clinical and echocardiographic parameters improves the prognostic stratification of patients across the HF spectrum.^7^ A more comprehensive, 5-site evaluation of congestion may further enhance diagnostic accuracy and refine risk stratification, potentially leading to more personalized and effective therapy and follow-up.^2^ NT-proBNP–guided therapy has failed to improve outcomes in both acute and chronic HF,^31,32^ whereas preliminary studies indicate that LUS-guided management significantly reduces urgent visits for worsening HF.^33–35^ Building on these observations, we propose a hierarchical, probability-informed algorithm for HF assessment (*Figure 3*). Routine clinical, laboratory, and echocardiographic data can be integrated to estimate the pre-test likelihood of congestion in patients at risk of or with established HF. Patients with a higher estimated likelihood can be prioritized for multi-organ US-based assessment, while those with a lower likelihood may undergo routine follow-up. This approach maintains US confirmation as the diagnostic gold standard while supporting individualized, resource-conscious management and facilitating early detection of subclinical congestion.

## Limitations

Although the cohort was prospectively enrolled, the analysis was observational; therefore, causal inferences cannot be drawn. The study was conducted at a single tertiary-care center within a dedicated dyspnea clinic, which may limit the generalizability of the findings and introduce selection bias toward a highly characterized population. From a methodological perspective, larger datasets are generally desirable for the development and validation of AI-driven models. In addition, hepatic and portal venous flow assessments were available only in subsets of patients, reflecting real-world feasibility constraints but potentially limiting the robustness of models involving these sites. The study was cross-sectional and did not include longitudinal assessment of congestion dynamics or hard clinical endpoints; therefore, the prognostic and therapeutic implications of AI-predicted congestion patterns remain to be established. Our findings should be interpreted as hypothesis-generating and further analyses—including external validation—are required before any quantitative application of the models can be proposed.

## Conclusions

AI-driven integration of multi-organ US with routinely available clinical and echocardiographic variables offers a novel, interpretable strategy for characterizing congestion across the HF spectrum. By capturing the multidimensional nature of fluid overload beyond traditional biomarkers, this approach offers a more nuanced and physiologically grounded interpretation of fluid overload across the HF spectrum and has the potential to improve phenotypic stratification and support more personalized clinical decision-making.

## Supplementary Material

qyag036_Supplementary_Data

## Data Availability

The data underlying this article will be shared on reasonable request to the corresponding author.

## Lead author biography

Nicola Riccardo Pugliese, MD, PhD, is an Assistant Professor of Cardiology at the University of Pisa and consultant cardiologist at Pisa University Hospital, Italy. His research focuses on advanced cardiovascular imaging and non-invasive phenotyping of HF across the full disease spectrum, with particular interest in multi-organ congestion, cardiopulmonary interactions, and interpretable artificial intelligence applied to imaging-derived data. He completed his medical and cardiology training at the University of Pisa and obtained a PhD in Clinical and Translational Sciences. He also attended the Postgraduate Course in Heart Failure Management (PCHF) of the European Society of Cardiology and the Heart Failure Association. His training includes research experience at leading international centers in cardiovascular imaging. Dr Pugliese holds multiple European certifications in echocardiography and vascular ultrasound and is a member of the Transthoracic Echocardiography Certification Committee of the European Association of Cardiovascular Imaging. He is actively involved in international collaborative research networks in heart failure and cardiovascular imaging.
