# Supplementary material for: Artificial intelligence-based characterization of multi-organ ultrasound congestion across the heart failure Spectrum
Source: Eur Heart J Imaging Methods Pract. 2026 Mar 4;4(1):qyag036. doi: 10.1093/ehjimp/qyag036 (PMC12975183; doi:10.1093/ehjimp/qyag036)
Supplement: qyag036_Supplementary_Data [file qyag036_supplementary_data.zip › Supplementary Appendix congestion AI.docx]

**SUPPLEMENTAL APPENDIX FOR**

**" Artificial Intelligence–Based Characterization of Multi-Organ Ultrasound Congestion Across the Heart Failure Spectrum"**

**Laboratory evaluation.** The estimated glomerular filtration rate (eGFR) was calculated using the Chronic Kidney Disease Epidemiology Collaboration formula^1^. NT-proBNP was measured with the ECLIA monoclonal assay using the Cobas e411 platform (Roche Diagnostics Italia, Monza, Italy). Serum concentrations of ferritin, transferrin, and iron were measured, and transferrin saturation (TSAT) was calculated using the formula: $\frac{iron}{transferrin x 25,2} x 100$.

**Baseline echocardiography.** All patients underwent a comprehensive transthoracic echocardiography examination according to international recommendations^2,3^. Stroke volume was calculated by multiplying the left ventricular (LV) outflow tract area by the LV outflow tract velocity-time integral measured by pulsed-wave Doppler. Cardiac output was calculated by multiplying stroke volume by heart rate. Inferior vena cava (IVC) diameter and its variations were used to estimate right atrial pressure (RAP), as recommended^2^. Systolic pulmonary artery pressure (sPAP) was measured from the peak tricuspid regurgitation velocity (TRV) with the simplified Bernoulli equation, adding the estimated RAP. The left atrial volume index (LAVi) was calculated with the disc summation algorithm (Simpson's technique) in a biplane approach from the apical four-chamber and two-chamber view^2^. Valvular regurgitation was qualitatively assessed using colour-Doppler, and whenever regurgitation was more than mild, it was quantified using the width of the vena contracta and the effective regurgitant orifice area^4^. All measurements were reported as the average of three beats for patients with normal sinus rhythm and five beats for patients with atrial fibrillation.

**Speckle tracking echocardiography (STE).** We measured LV global longitudinal strain (GLS) from the apical long-axis view and two- and four-chamber views, ensuring a frame rate >50 Hz (2D strain analysis, TomTec Imaging Systems, Unterschleissheim, Germany). We reported the average LV GLS values from the three apical views at rest. We excluded poorly tracked segments, and patients were not analyzed if more than one segment per view was deemed unacceptable. We measured the left atrial (LA) reservoir strain using the same software as the strain average in six segments in the four-chamber and two-chamber views, ensuring a frame rate >50 Hz. LA strain was measured using the QRS as the fiducial point. STE-derived measurements were reported as the average of three beats, and all measurements were performed offline by expert readers blinded to clinical and other instrumental data, according to our previously validated protocol^5,6^.

**Supplementary Table 1**. Population characteristics according to the American College of Cardiology/American Heart Association Heart Failure Staging System.

| **Variable** | **Stage A-B**  **(n=651)** | **Stage C-HFpEF**  **(n=561)** | **Stage C-HFrEF**  **(n=376)** |
| --- | --- | --- | --- |
| **DEMOGRAPHICS** |  |  |  |
| Age, years | 68 (56-77) | 78 (72-84) | 71 (60-79)* |
| Males | 424 (65) | 274 (49) | 302 (80)* |
| BMI, kg/m^2^ | 26 (24-29) | 26 (23-29) | 26 (24-29)* |
| Arterial hypertension | 419 (64) | 428 (76) | 222 (59)* |
| Diabetes mellitus | 140 (22) | 136 (24) | 116 (31)° |
| Dyslipidemia | 340 (53) | 336 (60) | 243 (65)° |
| History of atrial fibrillation | 57 (9) | 256 (46) | 152 (40)* |
| Atrial fibrillation/flutter | 13 (2) | 164 (31) | 96 (26)* |
| Stroke/TIA | 36 (6) | 46 (8) | 32 (9) |
| CAD | 69 (11) | 91 (16) | 171 (46)* |
| Coronary revascularisation | 65 (10) | 82 (15) | 149 (40)* |
| Previous MI | 38 (6) | 42 (8) | 141 (37)* |
| COPD | 19 (3) | 11 (2) | 18 (5)° |
| FEV1<50% | 6 (1) | 3 (1) | 2 (1) |
| **Clinical evaluation** |  |  |  |
| Heart rate, beat/min | 75 (65-85) | 75 (65-70) | 75 (65-75) |
| Systolic blood pressure, mmHg | 135 (125-146) | 130 (120-141)^a^ | 120 (110-135) |
| Diastolic blood pressure, mmHg | 80 (70-90) | 76 (70-85)^a^ | 75 (70-85)^a^ |
| Oxygen saturation, % | 98 (97-99) | 98 (97-98) | 98 (97-99) |
| NYHA classification |  |  |  |
| Class I | 430 (66) | 151 (27)° | 121 (32) |
| Class II | 176 (27) | 298 (53) | 195 (52) |
| Class III | 45 (7) | 112 (20) | 60 (16) |
| KCCQ score, % | 80 (65-89) | 68 (53-81)^a^ | 68 (66-81)^a^ |
| **Therapy** |  |  |  |
| Beta-blockers | 218 (34) | 409 (73) | 324 (86)* |
| DHP CCB | 155 (24) | 122 (22) | 37 (10)* |
| Non-DHP CCB | 11 (2) | 13 (2) | 2 (1) |
| Amiodarone | 6 (1) | 4 (6) | 16 (5)* |
| Digoxin | 0 (0) | 36 (6) | 37 (10)* |
| ACEi or ARB | 334 (51) | 343 (61) | 194 (51)* |
| MRA | 61 (9) | 180 (31) | 219 (58)* |
| ARNI | 4 (0.6) | 20 (4) | 132 (35)* |
| Loop diuretics | 98 (16) | 344 (61) | 274 (73)* |
| Thiazides/thiazide-like diuretics | 117 (18) | 22 (4) | 49 (13)* |
| SGLT2i | 22 (3) | 78 (14) | 84 (22)* |
| ICD | 8 (1) | 11 (2) | 113 (30)* |
| CRT | 0 (0) | 11 (2) | 69 (18)* |
| LAMA | 21 (3) | 25 (5) | 29 (8) |
| LABA | 31 (5) | 34 (6) | 31 (8) |
| Inhailed corticosteroids | 30 (5) | 33 (6) | 22 (6) |
| **BLOOD TESTS** |  |  |  |
| WBC, cells/µL | 6.16 (5.19-7.29) | 6.28 (5.13-7.53) | 6.43 (5.41-7.75)^c^ |
| NLR | 2.08 (1.66-2.85) | 2.52 (1.86-3.60)^a^ | 2.60 (1.83-3.52)^a^ |
| Hemoglobin, g/dL | 13.7 (12.7-14.5) | 12.7 (11.7-13.9)^a^ | 13.5 (12.4-14.6)^b^ |
| RDW, % | 13.3 (12.8-14.1) | 14.0 (13.2-15.2)^a^ | 14.1 (13.3-15.4)^a^ |
| TSAT, % | 23.6 (18.1-30.1) | 20.5 (14.8-26.1)^a^ | 20.8 (15.6-27.2)^c^ |
| Iron, 𝜇mol/L | 80 (64-98) | 72 (54-87)^a^ | 73 (54-97)^c^ |
| Ferritin, ng/mL | 118 (60-202) | 94 (49-182) | 105 (59-217) |
| Creatinine, mg/dL | 0.9 (0.8-1.1) | 1.0 (0.8-1.3)^a^ | 1.1 (0.9-1.4)^a,b^ |
| eGFR, mL/min/1.73 m^2^ | 77 (65-90) | 63 (47-78)^a^ | 65 (50-79)^a^ |
| ACR, mg/dL | 7.0 (3.3-18.5) | 12.7 (5.1-38.2)^a^ | 14.1 (5.6-59.3)^a^ |
| Na^+^, mEq/L | 141 (139-142) | 141 (139-142) | 140 (139-142) |
| K^+^, mEq/L | 4.3 (3.9-4.5) | 4.3 (4.0-4.7) | 4.3 (4.0-4.7) |
| Albumin, g/dL | 4.3 (3.8-4.5) | 4.2 (4.0-4.4) | 4.1 (3.8-4.4) |
| HbA1c, mmol/mol | 40 (37-48) | 41 (37-46)^a^ | 41 (37-48)^a^ |
| Total cholesterol, mg/dL | 170 (143-198) | 160 (133-185)^a^ | 147 (128-178)^a,d^ |
| LDL, mg/dL | 97 (75-121) | 89 (68-111)^a^ | 83 (59-110)^a^ |
| HDL, mg/dL | 53 (43-63) | 51 (43-61) | 45 (38-55)^a,b^ |
| Triglycerides, mg/dL | 91 (70-128) | 88 (71-118) | 97 (77-132)^d^ |
| Uric acid, mg/dL | 5.3 (4.3-6.3) | 5.5 (4.6-6.7)^c^ | 5.8 (4.7-7)^a^ |
| hs-CRP, mg/L | 0.14 (0.01-0.30) | 0.17 (0.02-0.38)^c^ | 0.17 (0.07-0.44)^c^ |
| NT-proBNP, pg/mL | 114 (60-204) | 830 (402-1647)^a^ | 1222 (492-2661)^a,b^ |
| hs-Troponin T, pg/mL | 12 (8-18) | 19 (12-31) | 22 (13-38) |

Values are n (%) or median (25^th^ quartile, 75^th^ quartile).

^a^ p<0.001 vs Stage A-B; ^b^ p<0.001 vs C-HFpEF; ^c^ p<0.05 vs Stage A-B, ^c^ p<0.05 vs Stage C-HFpEF

* p <0.001 for χ^2^ tests.

ACEi: angiotensin-converting enzyme inhibitor; ACR: albumin-to-creatinine ratio; AHA/ACC: American College of Cardiology/American Heart Association; ARB: angiotensin receptor blocker; ARNI: angiotensin receptor neprilysin inhibitor; BMI: body mass index; CAD: coronary artery disease; CRT: cardiac resynchronization therapy; DHP CCB: dihydropyridine calcium channel blocker; eGFR: estimated glomerular filtration rate; HbA1c: glycated hemoglobin; HFpEF: heart failure with preserved ejection fraction; HFrEF: heart failure with reduced ejection fraction; hs-CRP: high sensitivity C-reactive protein; hs-Troponin T: high sensitivity Troponin T; ICD: implantable cardioverter defibrillator; MI: myocardial infarction; MRA: mineralocorticoid receptor antagonist; NYHA: New York Heart Association; NLR: neutrophil-to-lymphocyte ratio NT-proBNP: N-terminal prohormone of brain natriuretic peptide; RDW: red cell distribution width; SGLT2i: sodium glucose co-transporter 2 inhibitors; TIA: transient ischemic attack, TSAT: transferrin saturation; WBC: white blood cell.

**Supplementary Table 2**. Baseline echocardiography according to the American College of Cardiology/American Heart Association HF Staging System.

| **Variable** | **Stage A-B**  **(n=651)** | **Stage C-HFpEF**  **(n=561)** | **Stage C-HFrEF**  **(n=376)** |
| --- | --- | --- | --- |
| **Left ventricle** |  |  |  |
| WMSI | 1 (1-1) | 1 (1-1) | 2 (2-2.2)^a,b^ |
| LVMi, g/m^2.7^ | 104 (86-121) | 110 (92-132) | 131 (109-158)^a,b^ |
| RWT | 0.39 (0.35-0.44) | 0.40 (0.35-0.44) | 0.30 (0.26-0.35)^a,b^ |
| LVEDVi, mL/m^2^ | 63 (52-75) | 65 (55-78) | 102 (82-121)^a,b^ |
| LV ejection fraction, % | 64 (59-68) | 62 (57-66)^a^ | 36 (30-44)^a,b^ |
| Average S', cm/s | 8.5 (7.2-10) | 6.5 (5.5-8)^a^ | 5.5 (4.5-6.5)^a,b^ |
| LV GLS, % | 17.5 (15.1-20.3) | 15.2 (12.8-18.1) ^a^ | 8.7 (6.3-10.8)^a,b^ |
| SV, mL/beat | 62.8 (51.2-77.2) | 56.5 (44.2-70.5)^a^ | 58.5 (48.0-74.7) |
| CO, L/min | 4.5 (3.5-5.6) | 4.5 (3.6-5.4) | 4.6 (3.7-5.9) |
| Mitral E, cm/s | 75 (65-95) | 100 (75-140)^a^ | 75 (55-110)^b^ |
| Mitral A, cm/s | 85 (68-105) | 94 (70-120)^c^ | 80 (55-100)^a,b^ |
| Mitral E/A | 0.89 (0.73-1.13) | 1.00 (0.72-1.45) | 0.88 (0.60-1.62) |
| Average E/e’ | 9 (7-11) | 12 (9-17) | 11 (8-16) |
| LA reservoir strain/E/e' | 3.32 (2.17-5.08) | 1.46 (0.83-2.36)^a^ | 1.30 (0.68-2.06)^a^ |
| **Left atrium** |  |  |  |
| LAVi, mL/m^2^ | 30.8 (24.4-38.2) | 43.8 (35-54.6)^a^ | 40.9 (32.4-56.4)^a^ |
| LA reservoir strain, % | 33 (25-40) | 19 (12-27)^a^ | 15 (11-22)^a,b^ |
| LA booster strain, % (SR only) | 16 (12-19) | 11 (7-15)^a^ | 8 (6-13)^a,c^ |
| **Right ventricle and pulmonary circulation** |  |  |  |
| RA area, cm^2^ | 19 (15-29) | 22 (17-29)^a^ | 22 (17-31)^a^ |
| TAPSE, mm | 22 (19-24) | 20 (18-22)^a^ | 18 (17-21)^a,b^ |
| sPAP, mmHg | 26 (22-33) | 39 (29-50)^a^ | 31 (25-43)^a,b^ |
| dPAP, mmHg | 8 (6-10) | 11 (8-15)^a^ | 11 (8-16)^a^ |
| TAPSE/sPAP, mm/mmHg | 0.79 (0.63-0.96) | 0.51 (0.38-0.71)^a^ | 0.59 (0.40-0.81)^a^ |
| TR severity |  |  |  |
| Mild | 592 (91) | 353 (63) | 290 (77)* |
| Moderate | 26 (4) | 112 (20) | 45 (12) |
| Severe | 7 (1) | 90 (16) | 38 (10) |
| EROA-TR^#^ | 0.29 (0.23-0.32) | 0.45 (0.31-0.62)^a^ | 0.39 (0.35-0.56)^a,b^ |
| **Congestion assessment** |  |  |  |
| IVC, mm* | 15 (15-18) | 17 (15-21)^a^ | 17 (15-20)^a^ |
| IVC ≥21 mm | 33 (5) | 135 (24) | 90 (16)* |
| IVC collapse <50% | 13 (2) | 90 (16) | 41 (11)* |
| B-lines | 1 (0-4) | 4 (2-11)^a^ | 5 (1-11)^a^ |
| B-lines≥4 | 117 (18) | 230 (41) | 154 (41)* |
| B-lines cardiogenic | 65 (10) | 168 (30) | 128 (34)* |
| RVF pattern |  |  |  |
| Continuous | 600 (92) | 377 (66) | 256 (68)* |
| Discontinuous: pulsatile | 47 (7) | 90 (16) | 56 (15) |
| Discontinuous: biphasic | 3 (1) | 39 (7) | 34 (9) |
| Discontinuous: monophasic | 0 (0) | 61 (11) | 30 (8) |
| Hepatic vein |  |  |  |
| S>D | 84 (71) | 48 (28) | 23 (33)* |
| S≤D | 31 (26) | 48 (28) | 11 (16) |
| Systolic flow reversal | 4 (3) | 75 (44) | 35 (51) |
| Portal vein pulsatility index | 21 (13-27) | 22 (13-47) | 21 (16-29) |
| Pulsatility index >30 | 25 (21) | 49 (37)° | 10 (26) |
| Multi-organ congestion |  |  |  |
| No US sign | 453 (53) | 220 (40) | 183 (48)* |
| 1 US sign | 165 (36) | 180 (32) | 113 (30) |
| ≥2 US signs | 33 (11) | 161 (28) | 80 (22) |

Values are n (%) or median (25^th^ quartile, 75^th^ quartile).

^a^ p<0.001 vs Stages A-B; ^b^ p<0.001 vs Stage C-HFpEF; ^c^<0.05 vs Stages A-B;

* p<0.001; ° p<0.05 for χ^2^ tests.

^#^ only in at least moderate tricuspid regurgitation

HVF was performed in n=359 and PVF in n=289

CO: cardiac output; EDVi: end-diastolic volume index; EROA: effective regurgitant orifice area; IVC: inferior vena cava; LA: left atrium: LAVi: left atrial volume index; LV: left ventricle; LVEDVi: left ventricle end-diastolic volume index; LV GLS: left ventricle global longitudinal strain; LVMi: left ventricular mass index; RVF: renal venous flow; RWT: relative wall thickness; sPAP: systolic pulmonary artery pressure; SR, sinus rhythm; SV: stroke volume; TAPSE: tricuspid annular plane systolic excursion; TR: tricuspid regurgitation; WMSI: wall motion score index.

**Supplementary Table 3.** List of the variables used as input for the AI-driven models.

| **Domain** | **Variable** | **Missing Count** |
| --- | --- | --- |
| **Demographics** |  |  |
|  | Age, years | 0 |
|  | Males | 0 |
|  | BMI, kg/m^2^ | 0 |
|  | Arterial hypertension | 0 |
|  | Diabetes mellitus | 0 |
|  | Dyslipidemia | 0 |
|  | Smoke | 0 |
|  | History of atrial fibrillation | 0 |
|  | Atrial fibrillation/flutter | 0 |
|  | Stroke/TIA | 0 |
|  | CAD | 0 |
|  | Coronary revascularisation | 0 |
|  | Previous MI | 0 |
|  | COPD | 0 |
|  | FVC, % predicted | 0 |
|  | FEV1, % predicted | 0 |
|  | Heart rate, beat/min | 0 |
|  | Systolic blood pressure, mmHg | 0 |
|  | Diastolic blood pressure, mmHg | 0 |
|  | Oxygen saturation; % | 0 |
|  | NYHA classification | 0 |
|  | KCCQ score, % | 0 |
|  | Beta-blockers | 0 |
|  | DHP CCB | 0 |
|  | Non-DHP CCB | 0 |
|  | Amiodarone | 0 |
|  | Digoxin | 0 |
| **Blood tests** |  |  |
|  | WBC, cells/µL | 32 |
|  | NLR | 36 |
|  | Hemoglobin, g/dL | 21 |
|  | Hematocrit, % | 21 |
|  | PLT, cells/µL | 46 |
|  | RDW, % | 30 |
|  | TSAT, % | 45 |
|  | Iron, 𝜇mol/L | 45 |
|  | Ferritin, ng/mL | 45 |
|  | Creatinine, mg/dL | 23 |
|  | Urea, mg/dL | 46 |
|  | ACR, mg/dL | 47 |
|  | Na+, mEq/L | 24 |
|  | K+, mEq/L | 25 |
|  | Albumin, g/dL | 79 |
|  | HbA1c, mmol/mol | 29 |
|  | Fasting blood glucose, mg/dL | 38 |
|  | Tot cholesterol, mg/dL | 31 |
|  | LDL, mg/dl | 31 |
|  | HDL, mg/dl | 31 |
|  | Triglycerids, mg/dL | 47 |
|  | Uric acid, mg/dL | 30 |
|  | Hs-CRP, mg/L | 28 |
|  | NT-proBNP, pg/mL | 27 |
|  | hs-Troponin T, pg/mL | 36 |
| **Echocardiography** |  |  |
|  | WMSI | 0 |
|  | LVMi, g/m^2.7^ | 0 |
|  | RWT | 0 |
|  | LVEDVi, mL/m^2^ | 0 |
|  | LV ejection fraction, % | 0 |
|  | Average S', cm/s | 15 |
|  | LV GLS, % | 37 |
|  | SV, mL/beat | 0 |
|  | CO, L/min | 0 |
|  | Mitral E, cm/s | 0 |
|  | Mitral A, cm/s | 265 |
|  | Mitral E/A | 265 |
|  | Average E/e’ | 0 |
|  | LAVi, mL/m^2^ | 0 |
|  | LA reservoir strain, % | 171 |
|  | LA booster strain, % (SR only) | 454 |
|  | LA reservoir strain/E/e' | 171 |
|  | RA area, cm^2^ | 305 |
|  | TAPSE, mm | 0 |
|  | sPAP, mmHg | 9 |
|  | dPAP, mmHg | 322 |
|  | TAPSE/sPAP, mm/mmHg | 9 |
|  | EROA-TR | 1491 |
|  | IVC, mm | 0 |
|  | IVC collapse <50% | 0 |
|  | B-lines | 0 |
|  | B-lines cardiogenic | 0 |
|  | RVF pattern | 0 |
|  | Hepatic vein | 1229 |
|  | Portal vein pulsatility index | 1299 |

Values are n.

**SUPPLEMENTARY FIGURE LEGENDS**

**Supplementary Figure 1. Performance of feature subset selection for Inferior Vena Cava.** Mean ± variance of log loss, macro-averaged F1-score (F1-macro), and one-vs-rest Receiver Operating Characteristic Area Under the Curve (ROC-AUC OVR) are shown for subset features (blue) and for using all features (red). Vertical dashed lines indicate the best-performing subset with input dimension ≤10 (blue) and the global best-performing subset across all dimensions (red). Shaded areas represent variability across runs.

**Supplementary Figure 2. Performance of feature subset selection for Lung Ultrasoun.** Mean ± variance of log loss, macro-averaged F1-score (F1-macro), and one-vs-rest Receiver Operating Characteristic Area Under the Curve (ROC-AUC OVR) are shown for subset features (blue) and for using all features (red). Vertical dashed lines indicate the best-performing subset with input dimension ≤10 (blue) and the global best-performing subset across all dimensions (red). Shaded areas represent variability across runs.

**Supplementary Figure 3.** **Performance of feature subset selection for Renal Venous Flow.** Mean ± variance of log loss, macro-averaged F1-score (F1-macro), and one-vs-rest Receiver Operating Characteristic Area Under the Curve (ROC-AUC OVR) are shown for subset features (blue) and for using all features (red). Vertical dashed lines indicate the best-performing subset with input dimension ≤10 (blue) and the global best-performing subset across all dimensions (red). Shaded areas represent variability across runs.

**Supplementary Figure 4.** **Performance of feature subset selection for Hepatic Venous Flow.** Mean ± variance of log loss, macro-averaged F1-score (F1-macro), and one-vs-rest Receiver Operating Characteristic Area Under the Curve (ROC-AUC OVR) are shown for subset features (blue) and for using all features (red). Vertical dashed lines indicate the best-performing subset with input dimension ≤10 (blue) and the global best-performing subset across all dimensions (red). Shaded areas represent variability across runs.

**Supplementary Figure 5.** **Performance of feature subset selection for Portal Venous Flow.** Mean ± variance of log loss, macro-averaged F1-score (F1-macro), and one-vs-rest Receiver Operating Characteristic Area Under the Curve (ROC-AUC OVR) are shown for subset features (blue) and for using all features (red). Vertical dashed lines indicate the best-performing subset with input dimension ≤10 (blue) and the global best-performing subset across all dimensions (red). Shaded areas represent variability across runs. The best performance reached by the model with ≤10 variables might be due to a smaller sample size, leading to overfitting.

**Supplementary Figure 6.** **Shapley additive explanations (SHAP)** **of the Inferio Vena Cava model.** Each panel shows the relationship between the feature value (x-axis) and its SHAP value (y-axis), indicating the feature’s contribution to the model output. Points are colored by the predicted probability of the positive class.

dPAP: diastolic pulmonary artery pressure; NT-proBNP: N-terminal prohormone of brain natriuretic peptide; sPAP: systolic pulmonary artery pressure; TAPSE: tricuspid annular plane systolic excursion.

**Supplementary Figure 7.** **Shapley additive explanations (SHAP)** **scatter plots of the Lung Ultrasound model.** Each panel shows the relationship between the feature value (x-axis) and its SHAP value (y-axis), indicating the feature’s contribution to the model output. Points are colored by the predicted probability of the positive class.

dPAP: diastolic pulmonary artery pressure; LA: left atrium; NT-proBNP: N-terminal prohormone of brain natriuretic peptide.

**Supplementary Figure 8.** **Shapley additive explanations (SHAP)** **scatter plots of the Renal Venous Flow model.** Each panel shows the relationship between the feature value (x-axis) and its SHAP value (y-axis), indicating the feature’s contribution to the model output. Points are colored by the predicted probability of the positive class.

dPAP: diastolic pulmonary artery pressure; LV SV: left ventricle stroke volume; LVMi: left ventricular mass index; NT-proBNP: N-terminal prohormone of brain natriuretic peptide; RDW: red cell distribution width; sPAP: systolic pulmonary artery pressure; TAPSE: tricuspid annular plane systolic excursion.

**Supplementary Figure 9.** **Shapley additive explanations (SHAP)** **scatter plots of the Hepatic Venous Flow model.** Each panel shows the relationship between the feature value (x-axis) and its SHAP value (y-axis), indicating the feature’s contribution to the model output. Points are colored by the predicted probability of the positive class.

NT-proBNP: N-terminal prohormone of brain natriuretic peptide; RDW: red cell distribution width; TAPSE/sPAP: tricuspid annular plane systolic excursion/ systolic pulmonary artery pressure; TSAT: transferrin saturation.

**Supplementary Figure 10.** **Shapley additive explanations (SHAP)** **of the Portal Venous Flow model.** Each panel shows the relationship between the feature value (x-axis) and its SHAP value (y-axis), indicating the feature’s contribution to the model output. Points are colored by the predicted probability of the positive class.

FVC: forced vital capacity; LA: left atrium; TAPSE/sPAP: tricuspid annular plane systolic excursion/ systolic pulmonary artery pressure; TSAT: transferrin saturation.

**Supplementary Figure 11. Model calibration and predicted probability distribution oft the 3-item model.** (A) Calibration curve showing the agreement between mean predicted probabilities and the observed fraction of positive cases; the dashed line represents perfect calibration, while the solid line represents the model performance. The model showed moderate calibration performance (Brier score of 0.072. (B) Distribution of predicted probabilities across the dataset, illustrating the spread and concentration of model outputs.
