# Supplementary material for: Artificial intelligence-based characterization of multi-organ ultrasound congestion across the heart failure Spectrum
Source: Eur Heart J Imaging Methods Pract. 2026 Mar 4;4(1):qyag036. doi: 10.1093/ehjimp/qyag036 (PMC12975183; doi:10.1093/ehjimp/qyag036)
Supplement: qyag036_Supplementary_Data [file qyag036_supplementary_data.zip › Supplementary Figure 11.pptx]

## Slide 1
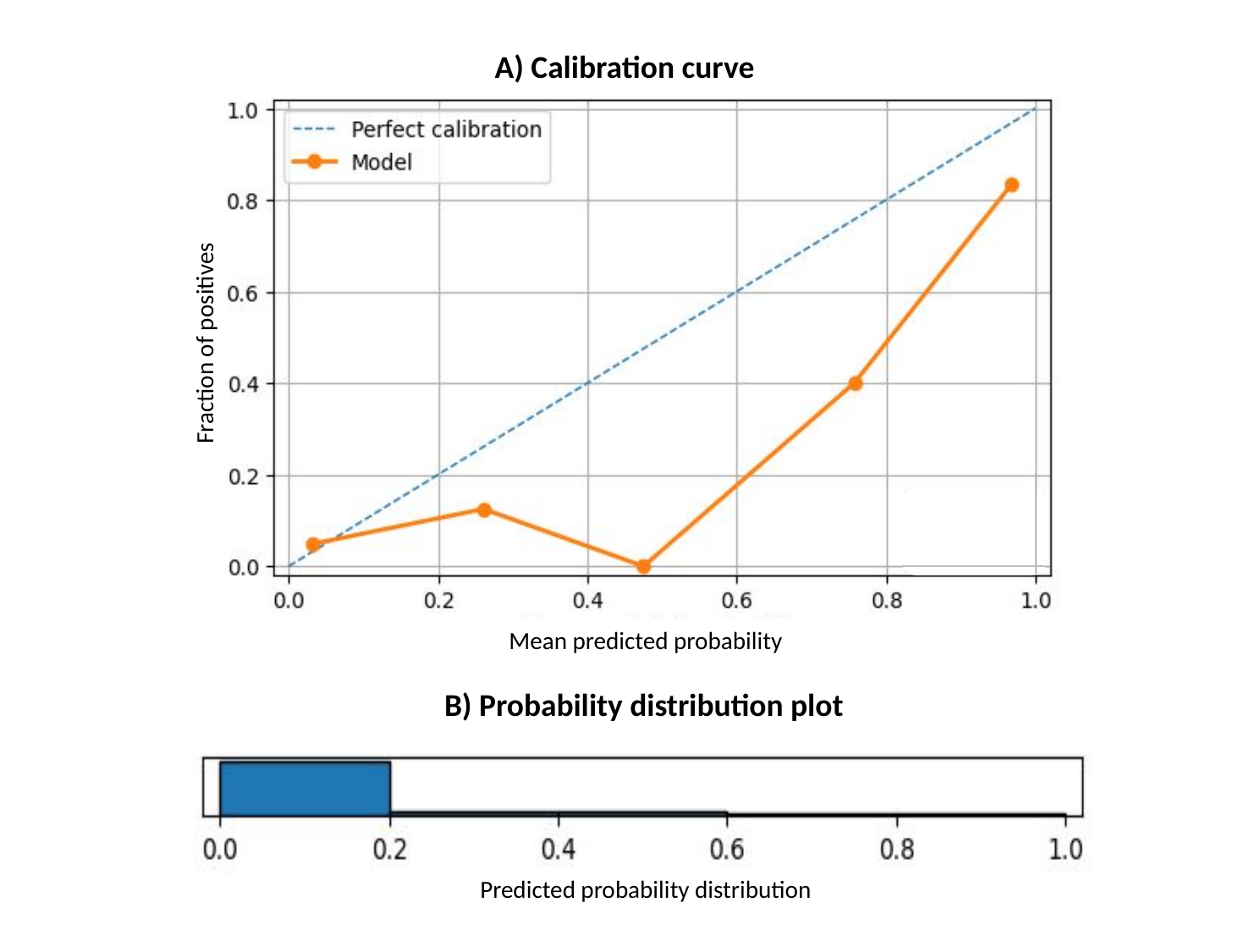

A) Calibration curve
Fraction of positives
Mean predicted probability
B) Probability distribution plot
Predicted probability distribution
