# Supplementary figures and images for: Artificial intelligence-based characterization of multi-organ ultrasound congestion across the heart failure Spectrum
Source: Eur Heart J Imaging Methods Pract. 2026 Mar 4;4(1):qyag036. doi: 10.1093/ehjimp/qyag036 (PMC12975183; doi:10.1093/ehjimp/qyag036)

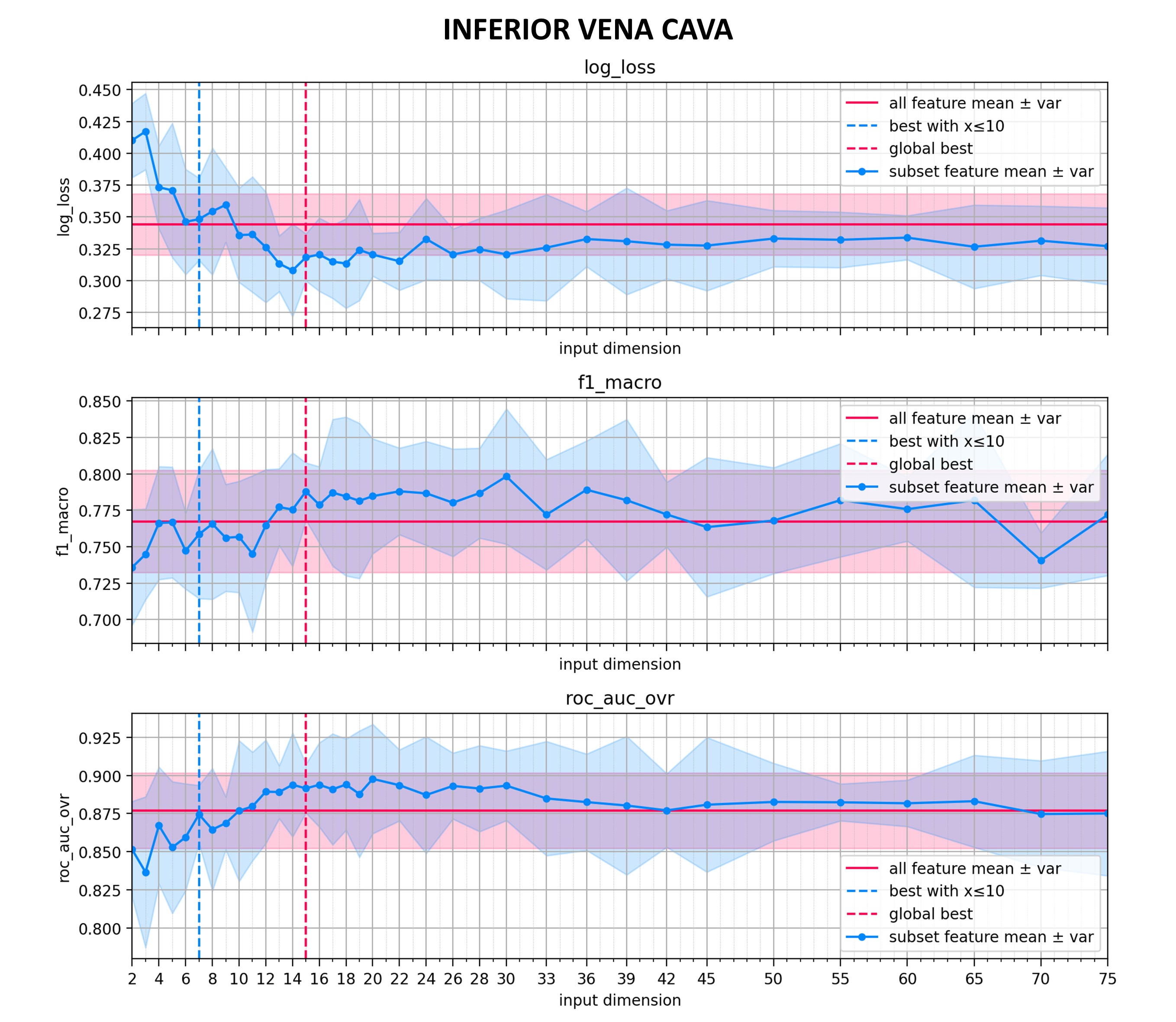

Supplement: qyag036_Supplementary_Data [file qyag036_supplementary_data.zip › Supplementary Figure 1.JPG]

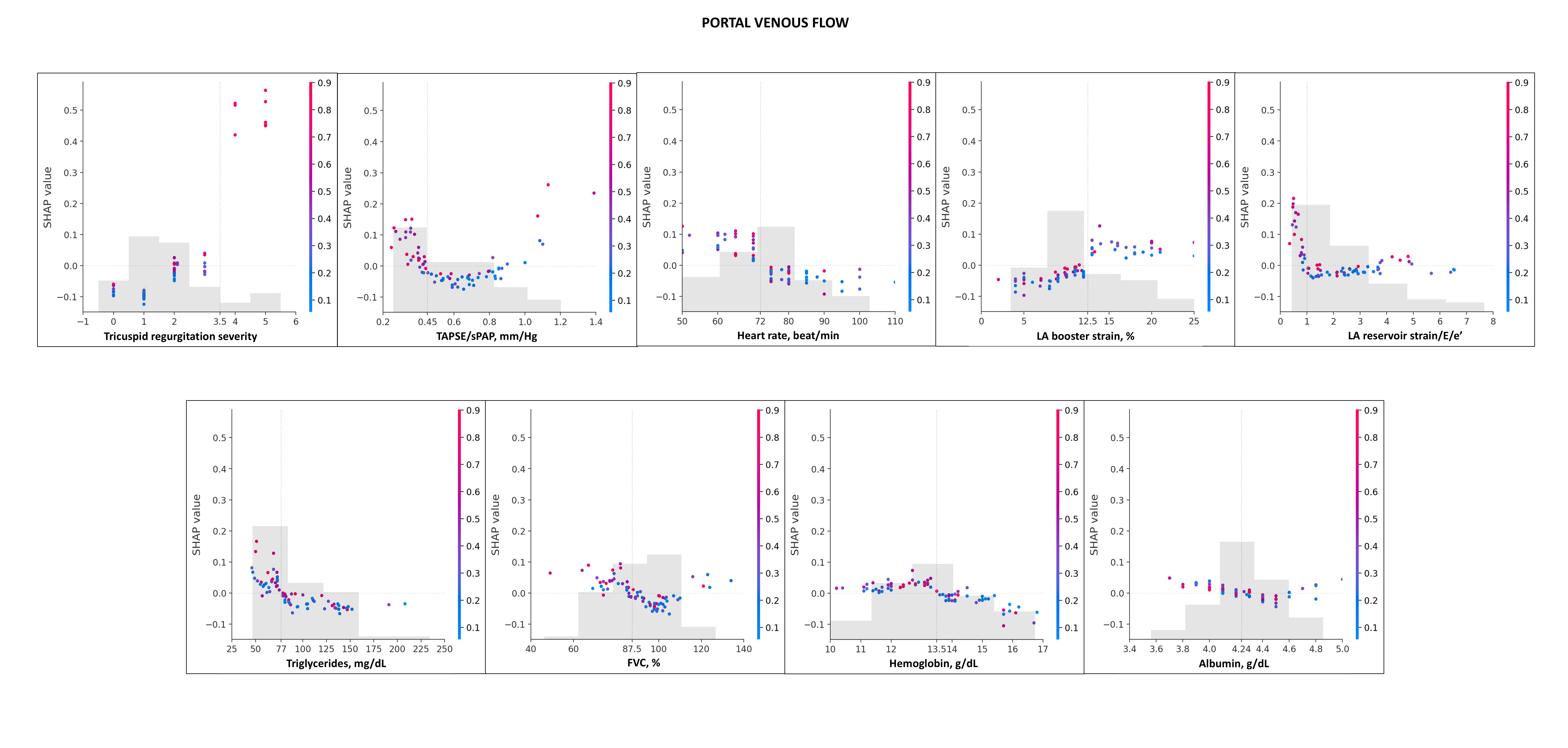

Supplement: qyag036_Supplementary_Data [file qyag036_supplementary_data.zip › Supplementary Figure 10.JPG]

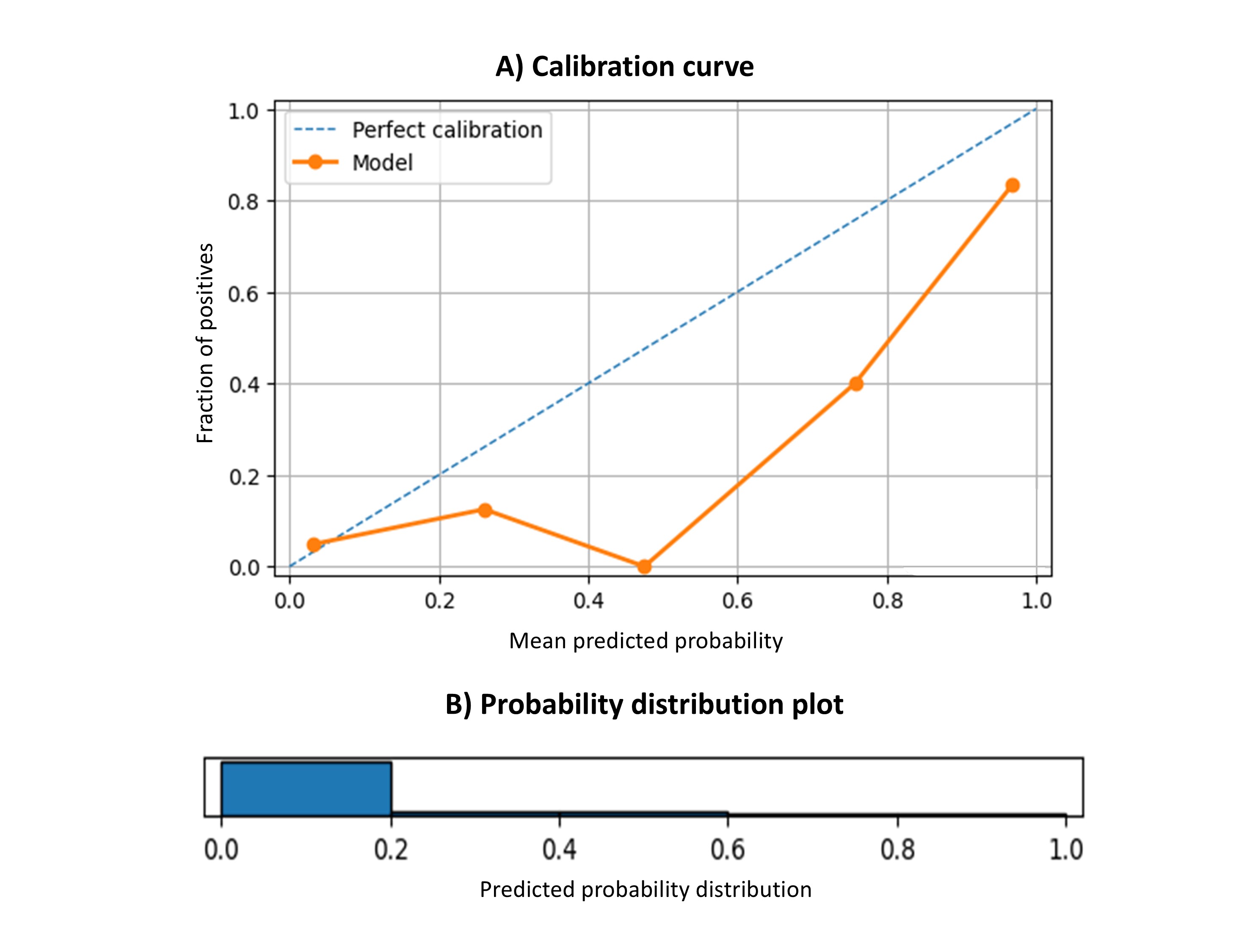

Supplement: qyag036_Supplementary_Data [file qyag036_supplementary_data.zip › Supplementary Figure 11.jpg]

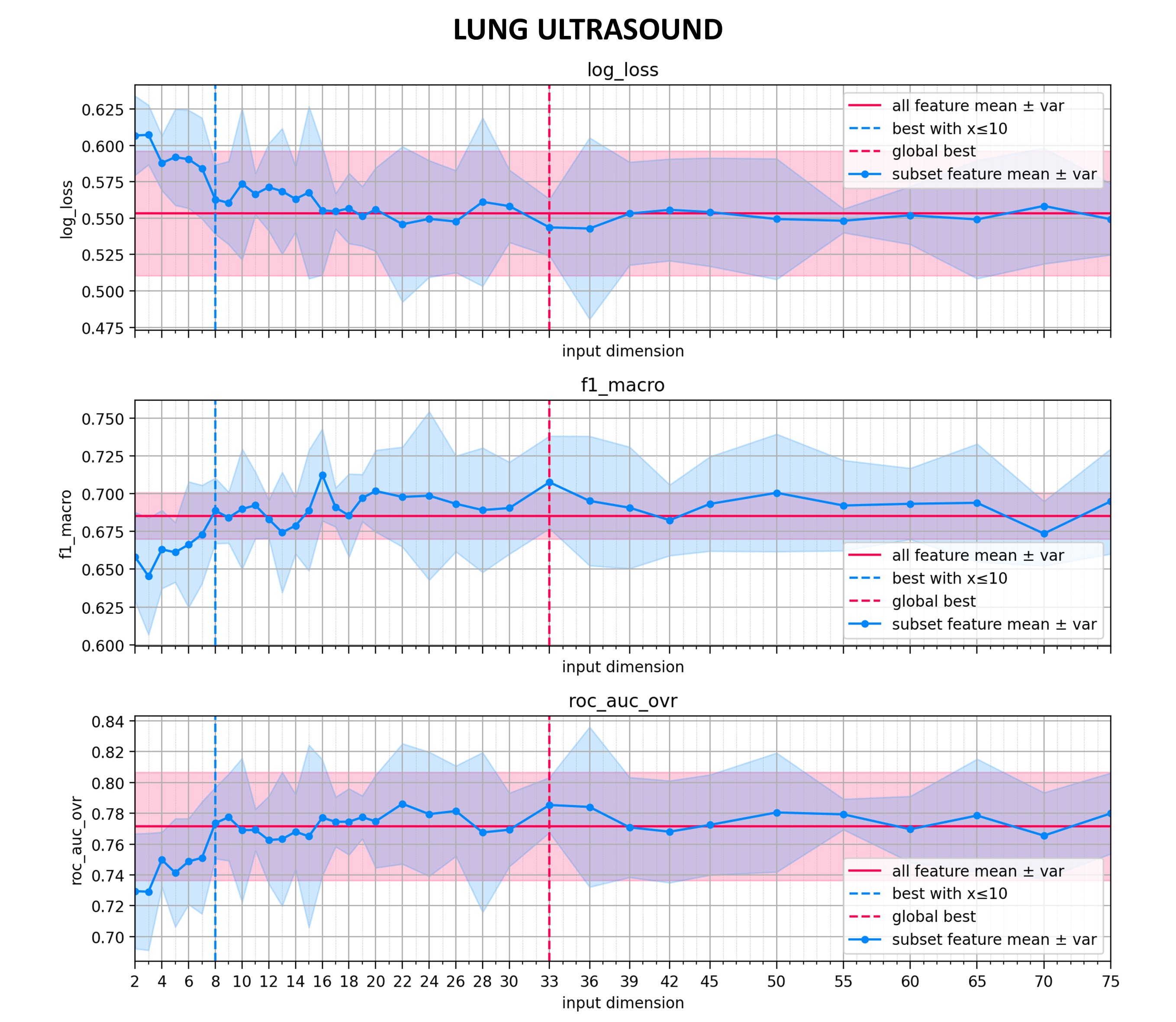

Supplement: qyag036_Supplementary_Data [file qyag036_supplementary_data.zip › Supplementary Figure 2.JPG]

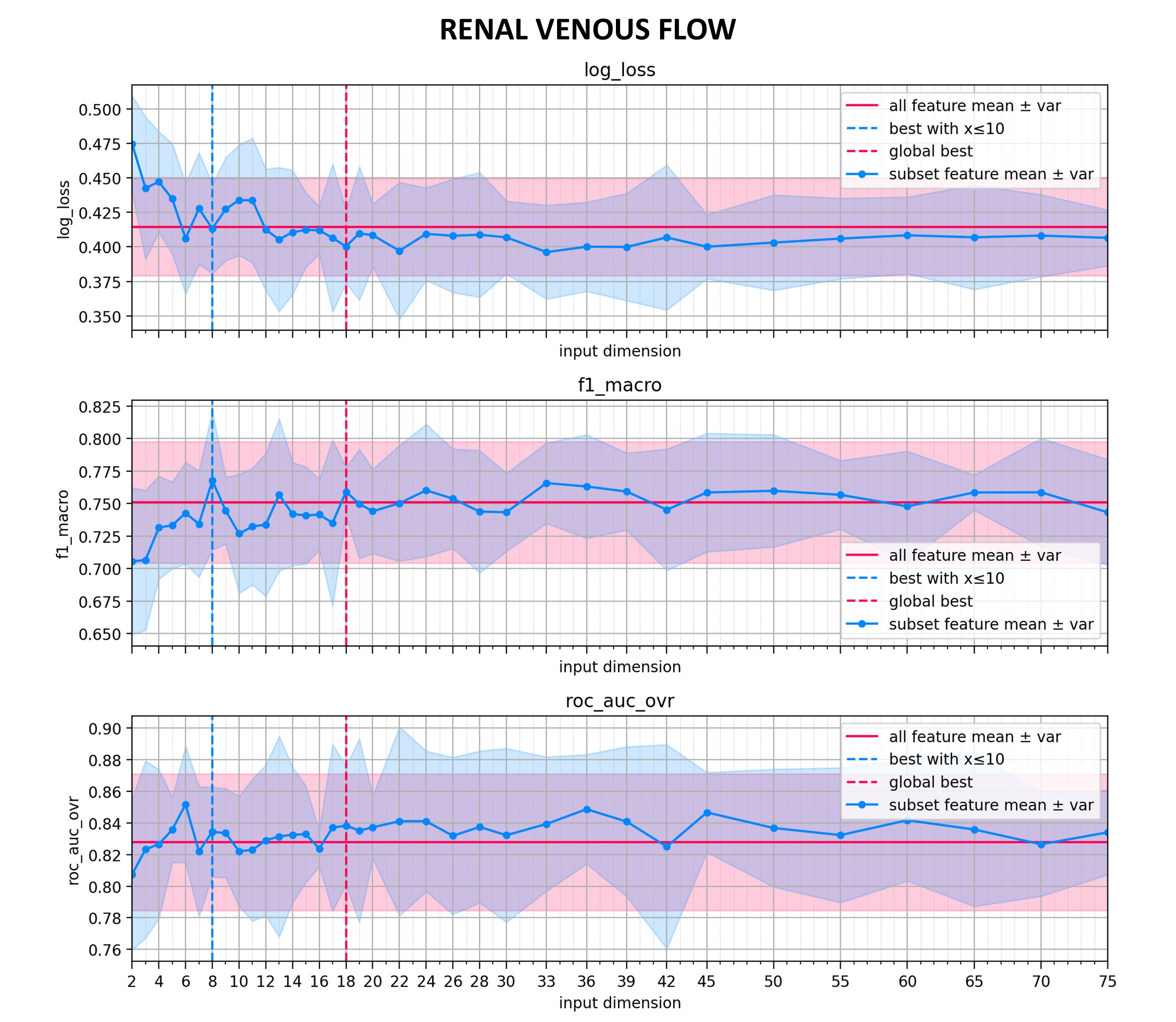

Supplement: qyag036_Supplementary_Data [file qyag036_supplementary_data.zip › Supplementary Figure 3.JPG]

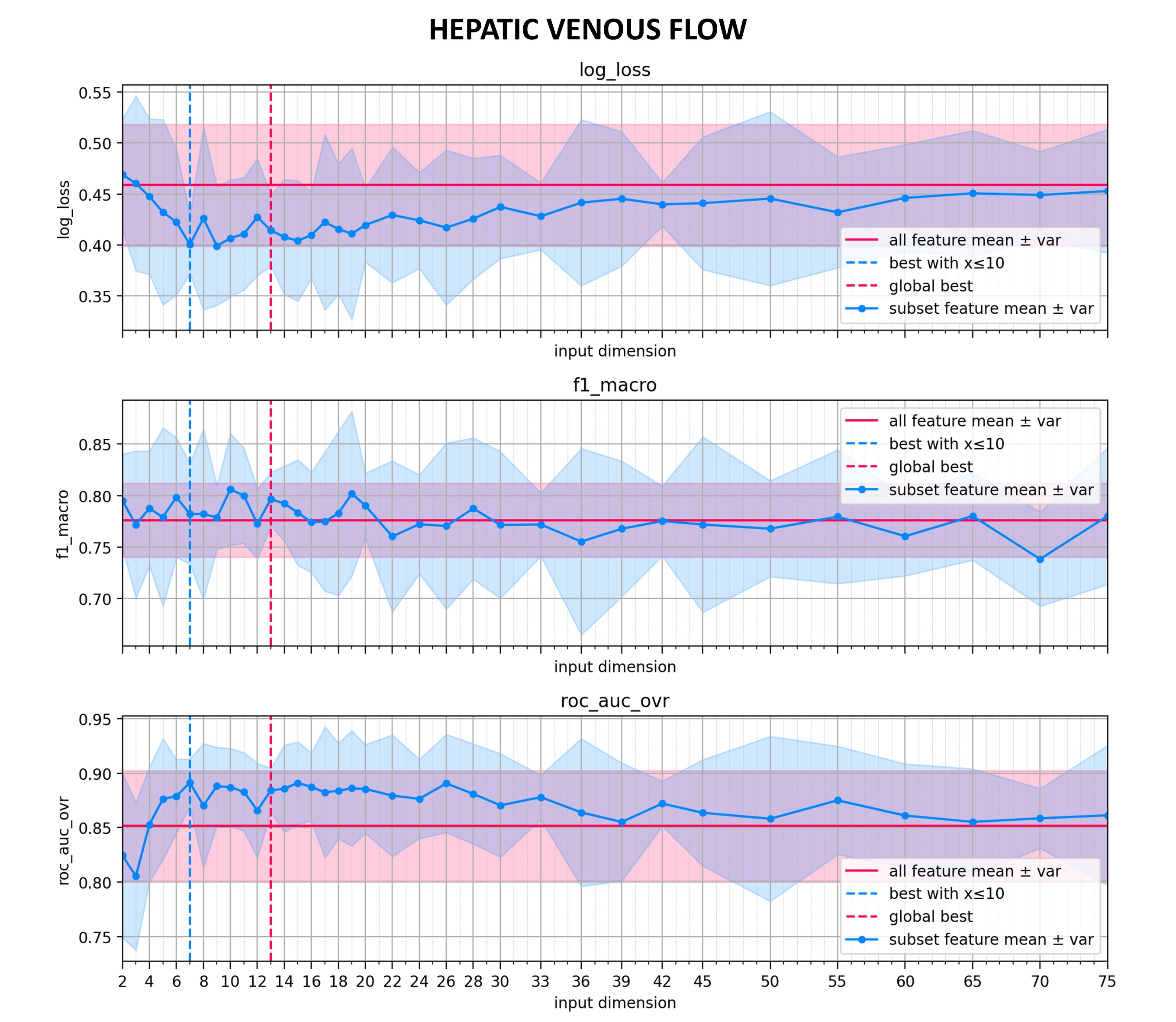

Supplement: qyag036_Supplementary_Data [file qyag036_supplementary_data.zip › Supplementary Figure 4.JPG]

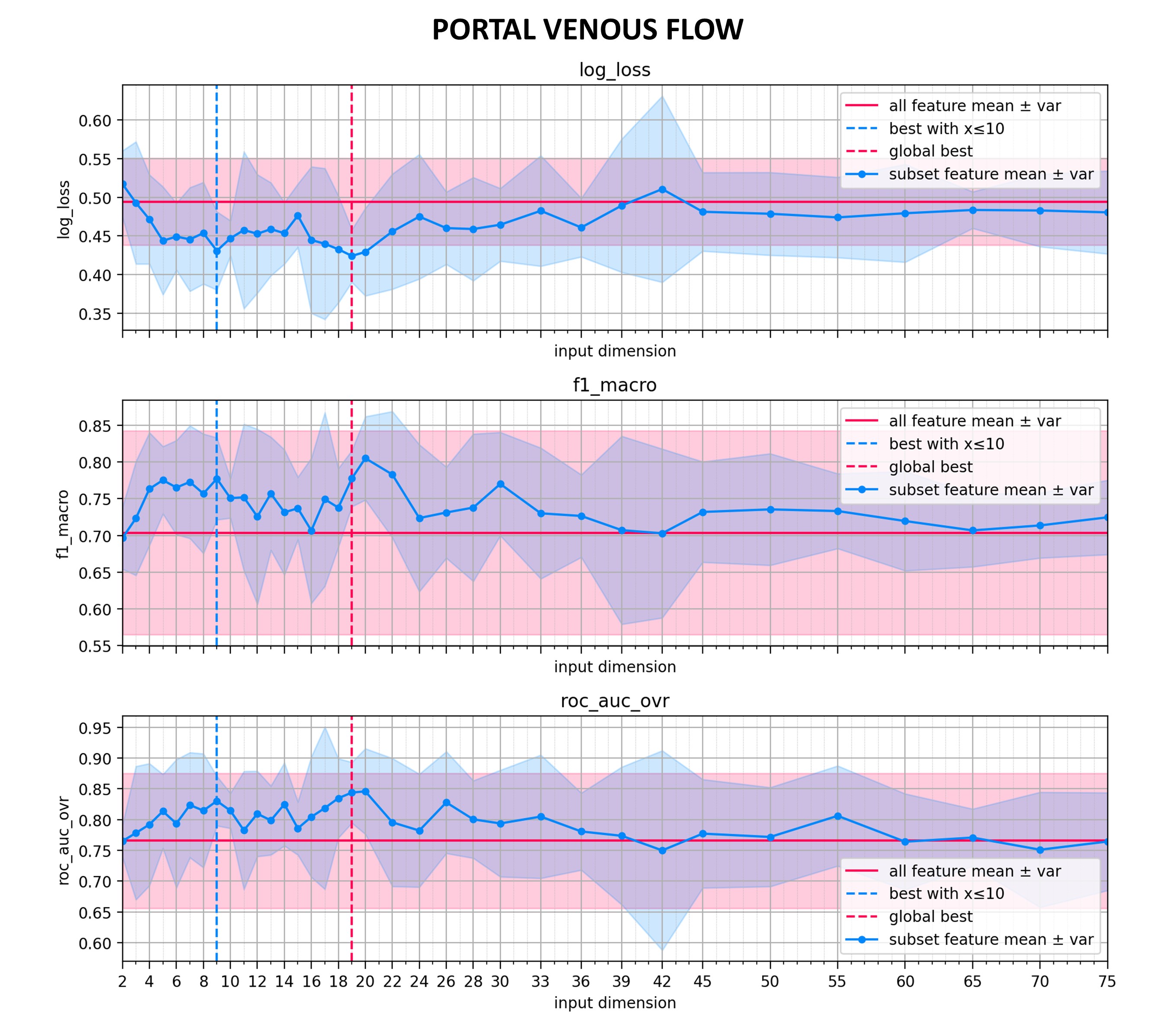

Supplement: qyag036_Supplementary_Data [file qyag036_supplementary_data.zip › Supplementary Figure 5.JPG]

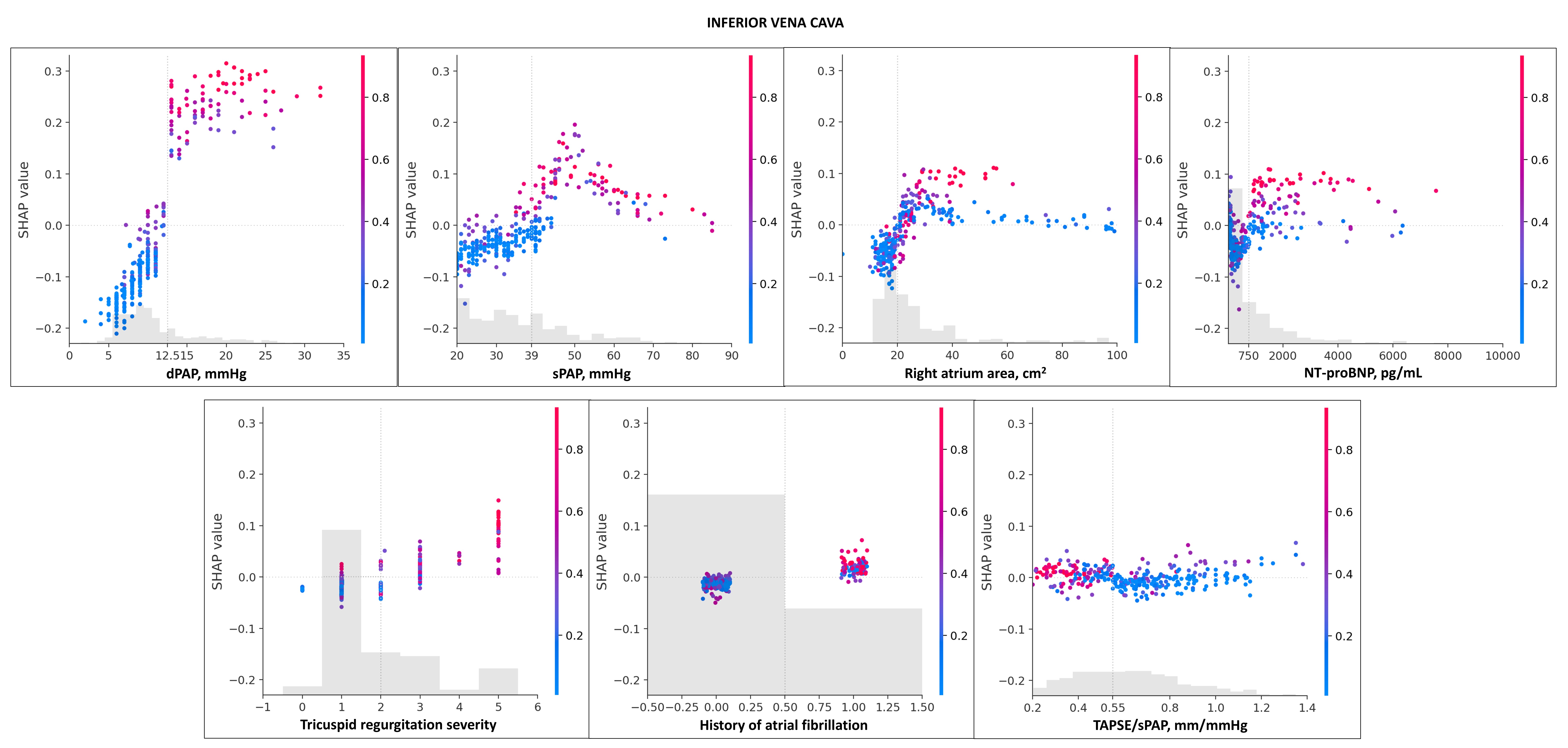

Supplement: qyag036_Supplementary_Data [file qyag036_supplementary_data.zip › Supplementary Figure 6.JPG]

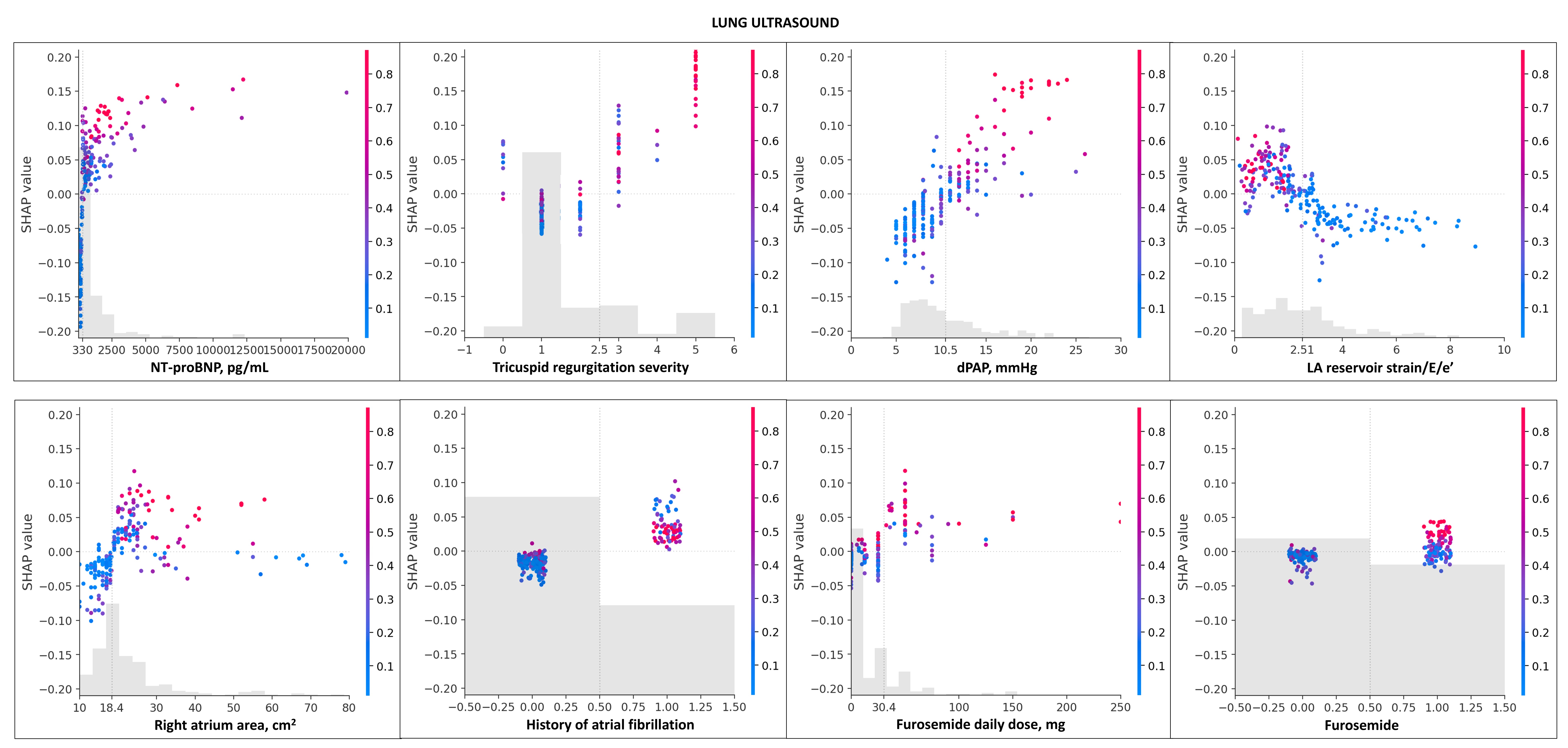

Supplement: qyag036_Supplementary_Data [file qyag036_supplementary_data.zip › Supplementary Figure 7.JPG]

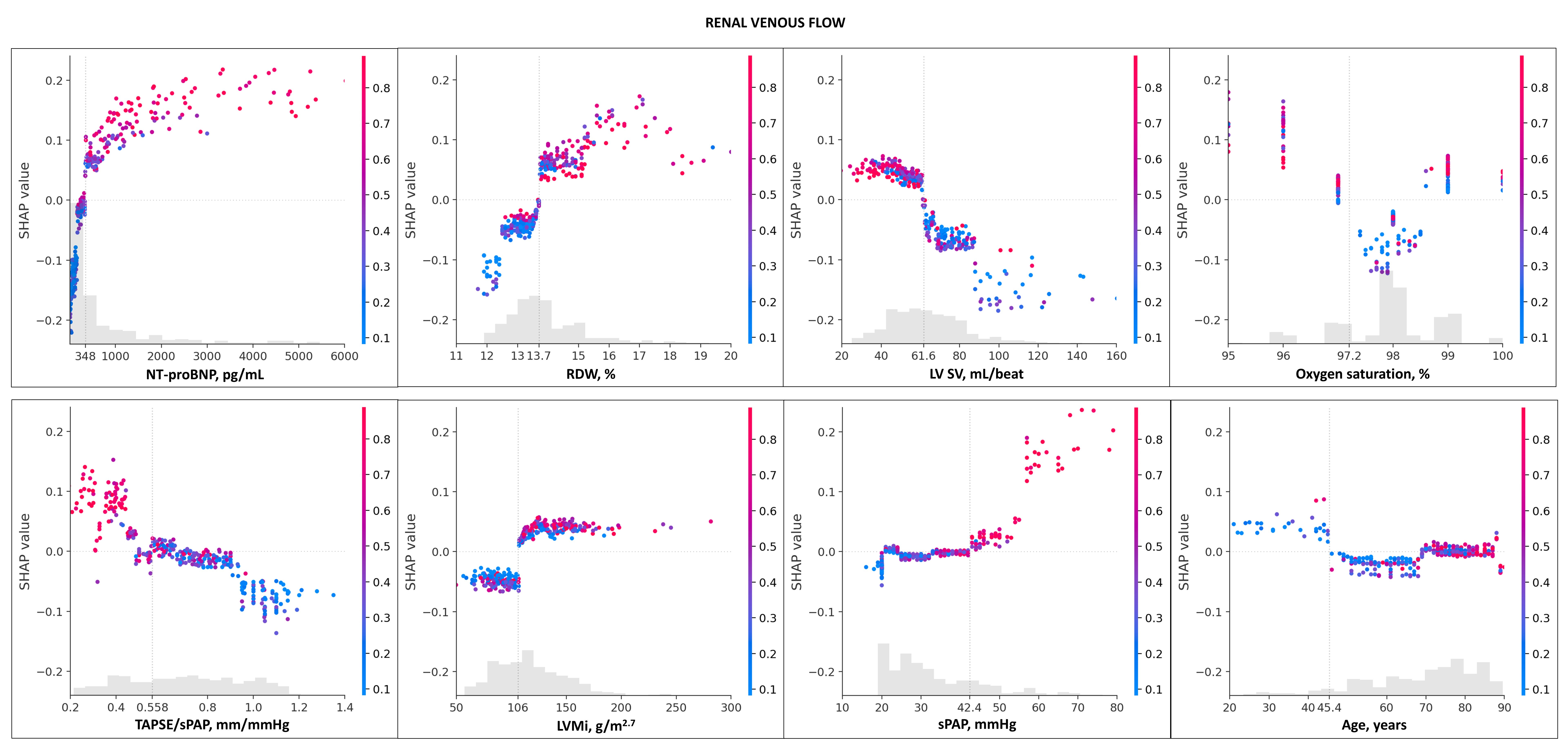

Supplement: qyag036_Supplementary_Data [file qyag036_supplementary_data.zip › Supplementary Figure 8.JPG]

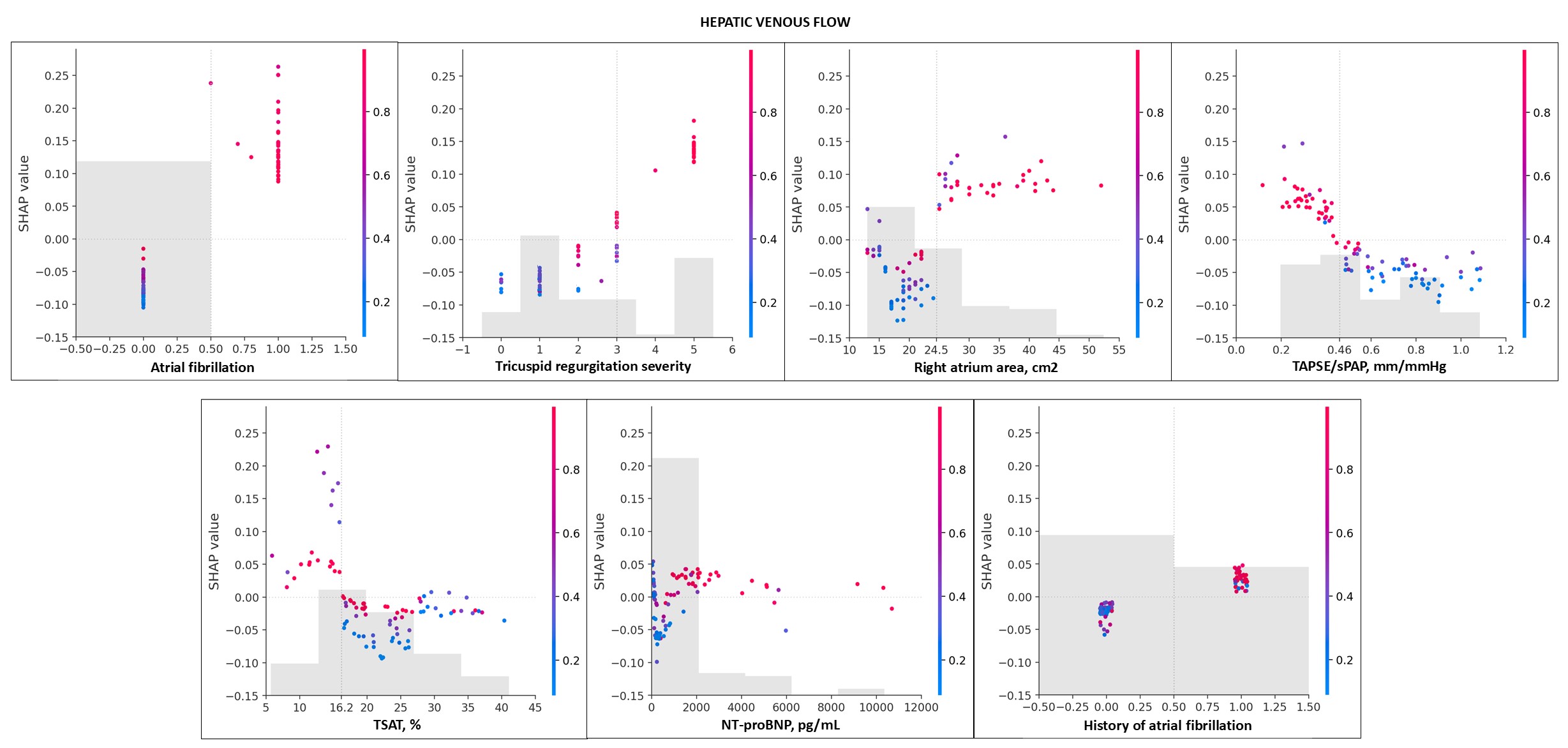

Supplement: qyag036_Supplementary_Data [file qyag036_supplementary_data.zip › Supplementary Figure 9.JPG]
